# Divergent Mast Cell Responses Modulate Antiviral Immunity During Influenza Virus Infection

**DOI:** 10.3389/fcimb.2021.580679

**Published:** 2021-02-19

**Authors:** Ashleigh R. Murphy-Schafer, Silke Paust

**Affiliations:** Department of Immunology and Microbiology, The Scripps Research Institute, La Jolla, CA, United States

**Keywords:** mast cells, influenza virus, IL-10 (interleukin-10), innate immunity, viral infection

## Abstract

Influenza A virus (IAV) is a respiratory pathogen that infects millions of people each year. Both seasonal and pandemic strains of IAV are capable of causing severe respiratory disease with a high risk of respiratory failure and opportunistic secondary infection. A strong inflammatory cytokine response is a hallmark of severe IAV infection. The widespread tissue damage and edema in the lung during severe influenza is largely attributed to an overexuberant production of inflammatory cytokines and cell killing by resident and infiltrating leukocytes. Mast cells (MCs) are a sentinel hematopoietic cell type situated at mucosal sites, including the lung. Poised to react immediately upon detecting infection, MCs produce a vast array of immune modulating molecules, including inflammatory cytokines, chemokines, and proteases. As such, MCs have been implicated as a source of the immunopathology observed in severe influenza. However, a growing body of evidence indicates that MCs play an essential role not only in inducing an inflammatory response but in suppressing inflammation as well. MC-derived immune suppressive cytokines are essential to the resolution of a number of viral infections and other immune insults. Absence of MCs prolongs infection, exacerbates tissue damage, and contributes to dissemination of the pathogen to other tissues. Production of cytokines such as IL-10 and IL-6 by MCs is essential for mitigating the inflammation and tissue damage caused by innate and adaptive immune cells alike. The two opposing functions of MCs—one pro-inflammatory and one anti-inflammatory—distinguish MCs as master regulators of immunity at the site of infection. Amongst the first cells to respond to infection or injury, MCs persist for the duration of the infection, modulating the recruitment, activation, and eventual suppression of other immune cells. In this review, we will discuss the immune modulatory roles of MCs over the course of viral infection and propose that the immune suppressive mediators produced by MCs are vital to minimizing immunopathology during influenza infection.

## Introduction

Influenza A virus (IAV) infects millions of people each year, causing respiratory disease ranging from mild to life-threatening. Annually, severe influenza causes the deaths of between 250,000 and 650,000 people globally (Iuliano et al., 2018; Krammer et al., 2018). Young children (under the age of 2), adults over 65 years or age, and people who are immunocompromised or have underlying chronic health conditions such as heart disease and diabetes are most vulnerable to developing and dying of severe influenza and associated complications (Adlhoch et al., 2019). The segmented RNA genome of IAV mutates rapidly in a process called antigenic drift, necessitating the annual reformulation of the seasonal influenza vaccine (Krammer et al., 2018). In addition, genetic reassortment between strains in a host simultaneously infected with multiple influenza serotypes produces novel IAV strains with pandemic potential (Webster et al., 1992; Krammer et al., 2018). Given increasing global human, livestock, and poultry population densities, the risk of zoonotic transmission of a highly-pathogenic, antigenically-distinct strain of IAV and subsequent global pandemic remains high (Fournie et al., 2013; Vincent et al., 2014; Krammer et al., 2018). Four such pandemics have occurred in the last century, resulting from reassortment of avian or swine influenzas to which humans lacked prior exposure (Lindstrom et al., 2004; Smith et al., 2009; Krammer et al., 2018). Due to the diversity of IAV strains across species, predicting the genetic makeup of future pandemic strains and preparing a broadly-neutralizing vaccine has proven difficult, though the development of antibodies targeting cross-clade conserved epitopes shows promise (Kwong et al., 2020). Antiviral treatments for influenza infections are limited, and development of viral resistance has rendered classes of antiviral drugs, such as adamantanes, unusable (Gubareva et al., 2010; Dong et al., 2015; Krammer et al., 2018). Antivirals currently used in the clinic, such as oseltamivir and zanamivir, have been shown to have limited efficacy in cases of severe influenza and are most efficient when administered within 48 h of symptom onset (Muthuri et al., 2014; Okoli et al., 2014; Dobson et al., 2015; Venkatesan et al., 2017). In light of the challenges to designing vaccines and therapeutics against a constantly evolving target such as IAV, development of new therapeutics effective across a broad range of influenza strains is urgently needed. One potential therapeutic alternative is boosting the host immune system to defend against the invading pathogen and minimize the tissue pathology associated with severe disease.

Extensive cytokine production in the lung is a hallmark of severe influenza infection (**Table 1**). IAV primarily infects and replicates in the pulmonary epithelial cells of the upper respiratory tract; in severe cases of IAV, the virus may reach the lower respiratory tract, replicating in alveolar epithelial cells and macrophages (Denney and Ho, 2018). Widespread epithelial cell death from both necrosis and apoptosis has been observed in the lungs of humans and mice during severe influenza infection (Short et al., 2014; Atkin-Smith et al., 2018). Recognition of the viral replication complex by the pattern recognition receptor (PRR) ZBP1/DAI triggers formation of the necrosome and cell death by necrosis, while the viral protein PB1-F2 induces the intrinsic pathway of apoptosis and the nonstructural viral protein NS1 contributes to upregulation of pro-apoptotic hallmarks of the extrinsic apoptosis pathway (Chen et al., 2001; Lam et al., 2008; Atkin-Smith et al., 2018; Fujikura and Miyazaki, 2018). Interestingly, NS1 also inhibits apoptosis through binding of pro-apoptotic proteins, suggesting the NS1 may play a role in delaying apoptosis to increase viral propagation (Obenauer et al., 2006; Liu et al., 2010; Adlhoch et al., 2019). The exact mechanisms dictating the specific fate of an infected cell are not yet fully elucidated, however, and may be strain dependent. The death of infected lung epithelial cells disrupts the integrity of the epithelial-endothelial barrier, allowing fluid accumulation in the lung and causing respiratory failure (Short et al., 2014). Secretion of inflammatory cytokines, type 1 interferons (IFNs), chemokines, and antimicrobial peptides by infected epithelial cells and macrophages recruits innate immune cells to the site of infection (**Table 1**) (Chan et al., 2005; Chan et al., 2010a; Chan et al., 2010b; Lam et al., 2010; Sanders et al., 2011; Xing et al., 2011; Denney et al., 2018). Activated innate immune cells, including macrophages, dendritic cells (DCs), neutrophils, mast cells (MCs), and natural killer (NK) cells, produce additional cytokines, contributing to the antiviral inflammatory milieu, often referred to as the “cytokine storm” (Ramos and Fernandez-Sesma, 2015; Liu et al., 2016). Adaptive immune cells, such as CD8+ cytotoxic T lymphocytes (CTL) and CD4+ T helper (Th) cells, also contribute to the highly inflammatory environment, a state which can persist even after the virus has been largely cleared from the lung. MCs, in particular, are major producers of cytokines and chemokines in the lung throughout the course of influenza infection. MC-derived cytokines and chemokines recruit and activate local and circulating innate and adaptive immune cells as well as regulate the composition, distribution, and phenotype of the structural and non-immune cells in the vicinity (Baram et al., 2001; Murray et al., 2004; Krystel-Whittemore et al., 2015; Reber et al., 2015; Oldford et al., 2018). To better understand how to minimize inflammatory immunopathology while still clearing the infection, attention has turned to better understanding how the immune system self-regulates inflammation. In this review, we will address the role of MCs in modulation of the inflammatory environment of the influenza-infected lung.

**Table 1:** Cytokine and chemokine expression by resident lung cells during influenza virus infection.

Human			
Strain	Cell type	Biological observations*	Reference
H1N1	Primary monocytes	Secretion of MCP-1 (CCL2), MIP-1α (CCL3), and RANTES (CCL5)	Sprenger et al. (1996)
H1N1 H5N1	Primary bronchial epithelial cells	Secretion of RANTES (CCL5) and IFNβ	Chan et al. (2010b)
H1N1 H5N1 H9N2	Bronchial epithelial cell line NCI-H292	Strain-dependent differential and temporal upregulation of TNF-α, IL-6, IL-8, CCL5, and CXCL10	Lam et al. (2010)
H3N2	Primary nasal and bronchial epithelial cells	Secretion of RANTES (CCL5)	Matsukura et al. (1998)
H9N2	Primary tracheobronchial epithelial cells	Upregulation of IFNβ, IP-10, and CCL5	Xing et al. (2011)
**Mouse**			
**Virus**	**Cell type**	Biological observations*	**Reference**
H1N1	Primary Balb/C alveolar epithelial cells	Upregulation of ICAM-1, VCAM-1, integrin-associated protein (IAP), CCL2, and CCL5	Herold et al. (2006)

*Relative to controls.

MCs are a lineage of innate immune hematopoietic sentinel cells, dispersed throughout the body but found most concentrated at the interfaces between the host and the environment, such as the skin, gut, and mucosa (Kitamura et al., 1977; Galli et al., 2008; Da Silva et al., 2014; Reber et al., 2015). The positioning of MCs at mucosal sites allows them to rapidly respond to infection and injury (Galli et al., 2008; Reber et al., 2015). Within minutes, activation of MCs can trigger the immediate release of preformed granules, which contain an array of proteases (such as tryptase and chymase), amines (such as histamine), and cytokines (Riley, 1953; Pejler et al., 2010; Caughey, 2011; Gri et al., 2012; Reber et al., 2015). Additional immune mediators, including cytokines, chemokines, and growth factors, are produced and secreted in an activation-dependent manner within hours and can continue for days (Da Silva et al., 2014; Sibilano et al., 2014; Reber et al., 2015). MCs express multiple Fc receptors on the cell surface, allowing MCs to respond to antigens previously encountered by the host (Galli et al., 2008; Krystel-Whittemore et al., 2015). The majority of Fc receptors on MCs are FcϵR1, the high affinity IgE receptor. Binding of IgE to FcϵR1 sensitizes the MCs, and polyvalent crosslinking of the target antigen initiates granule release and cytokine production. In addition to Fc receptors, MCs express an abundance of activating receptors on the cells surface and intracellularly, including pattern-recognition receptors (PRRs), such as Toll-like receptors (TLRs) and retinoic-acid inducible gene 1-like receptors (RLRs), complement receptors, CD48, and integrins (Sandig and Bulfone-Paus, 2012; Reber et al., 2015). PRR expression is not uniform across mast cell populations, however, and varies both with the tissue localization of the MCs and activating stimuli present (Saluja et al., 2012; Sandig and Bulfone-Paus, 2012; Agier et al., 2016; Akula et al., 2020; Plum et al., 2020). The diversity of activating receptors expressed by MCs make these cells versatile and capable of prolonged activation, able to release multiple waves of mediators, which vary with time, stimulus, and cellular environment (Gri et al., 2012).

MCs have long been associated with allergic responses and asthma; however, an important niche for MCs in host defense against infection and injury has been established (Rathore and St John, 2020). MCs play a protective role in a number of viral, bacterial, and parasitic infections, and mitigate tissue damage caused by exposure to animal venoms and toxins (Abraham and St John, 2010; Reber et al., 2015; Marshall et al., 2019). In addition to producing an array of cytokines and chemokines, including type 1 and 3 IFNs, IL-1α, IL-1β, IL-4, IL-6, IL-8, IL-10, IL-17, IL-33, TNF-α, TGF-β, SCF, CCL2, CCL3, and CCL5, MCs produce proteases chymase and tryptase capable of degrading toxic peptides and venoms (Metz et al., 2006; Akahoshi et al., 2011; Gri et al., 2012). MCs have been found to phagocytose a number of gram-positive and gram-negative bacteria including *Escherichia coli*, *Klebsiella pneumoniae, and Mycobacterium tuberculosis*, releasing TNF-α and reactive oxygen species in response (Abraham and Malaviya, 1997; Arock et al., 1998; Munoz et al., 2009; Abraham and St John, 2010). As tissue resident immune cells, MCs are uniquely poised to respond immediately at the site of infection or injury and persist through the duration of the infection or wound healing—inducing a pro-inflammatory environment, remodeling the cellular architecture, activating tissue resident immune cells, and recruiting additional immune responders from the periphery (Abraham and St John, 2010; Reber et al., 2015; Marshall et al., 2019).

Thorough investigations of the MC pro-inflammatory cytokine profile during viral infections have been published (Graham et al., 2015; Marshall et al., 2019). However, the presence of anti-inflammatory or immune suppressive cytokines of MC origin at the site of infection also suggests that MCs play a role in the “ramp down” of the immune response as infection is cleared (Galli et al., 2008; Reber et al., 2015). Two such cytokines are IL-10 and IL-6, which have been shown to be powerful mediators of inflammation during influenza infection (Sun et al., 2009; Yu et al., 2011; Yang et al., 2017). IL-10 is the founding member of the IL-10 family of structurally similar cytokines that also includes IL-19, IL-20, IL-22, IL-24, IL-26, and the type III IFNs (Ouyang et al., 2011). IL-10 binds the cell surface receptors IL-10R1 and IL-10R2 which heterodimerize, activating the STAT3 signaling pathway and inhibiting production of pro-inflammatory cytokines (Kotenko et al., 1997; Takeda et al., 1999; Walter, 2014). IL-6 is a pleotropic cytokine often associated with strong inflammatory responses but that can function as a powerful anti-inflammatory mediator as well (Hunter and Jones, 2015; Jones and Jenkins, 2018). All members of the IL-6 family of cytokines signal through the membrane glycoprotein gp130 (Silver and Hunter, 2010). IL-6 signaling requires binding to soluble or membrane-bound IL-6Rα which, in turn, interacts with gp130 (Jones et al., 2001; Hunter and Jones, 2015). Homodimerization of IL-6:IL-6Rα-bound gp130 activates the STAT3 signaling pathway (Heinrich et al., 2003). While IL-6Rα expression is confined predominantly to leukocytes and hepatocytes, gp130 is universally expressed (Hunter and Jones, 2015). Interactions in *trans* between gp130 and membrane-bound or soluble IL-6:IL-6Rα complexes allows IL-6 to signal to a range of cell types that are otherwise unresponsive to IL-6 and contributes to the diversity of downstream effects triggered by IL-6 signaling (Peters et al., 1996; Hunter and Jones, 2015; Reeh et al., 2019). In this review, we will address the mechanisms by which MCs suppress the immune response as infection or injury resolves and discuss a potential role MCs in mitigating inflammatory tissue damage in influenza-infected lungs.

## Mast Cell Recruitment to the Lung During Influenza A Virus Infection

MCs accumulate in the lungs of mice infected with both seasonal and pandemic strains of influenza (Josset et al., 2012; Morrison et al., 2014; Zarnegar et al., 2017). Lung resident MCs are likely amongst the first cells to respond to the presence of influenza in the lungs, and MCs progenitors (MCps) in the lung proliferate during influenza infection. However, the majority of the increase in MC population in the upper airways appears to derive from MCps recruited from the blood (Zarnegar et al., 2017). Respiratory virus infection of lung epithelial cells induces expression of the vascular cell adhesion molecule-1 (VCAM-1) which is required for the recruitment and infiltration of circulating MCps into the infected lung (Wang et al., 2000; Abonia et al., 2006; Zarnegar et al., 2017). These recruited progenitors proliferate rapidly after infection and remain in lung tissue up to three weeks post-infection, a time point at which most inflammation the lung has resolved. Recruitment and proliferation of MCps has also been detected in the lung tissue of children with viral lower respiratory tract infections such as severe influenza (Andersson et al., 2018).

A number of cytokines and chemokines have been identified to play a substantial role in MCp recruitment, although none have been identified as essential. For example, administration of the TLR3-agonist PolyI:C or the cytokine IL-33 induces moderate MCp recruitment to the lungs (Zarnegar et al., 2018). However, influenza infection of *Tlr3^-/-^* or IL-33 receptor *Il1rl1^-/-^* mice showed no reduction in MC progenitor recruitment. Influenza-infected alveolar macrophages and pulmonary epithelial and endothelial cells produce a number of additional cytokines and chemokines that are known MCp attractants (**Table 1**) (Sprenger et al., 1996; Matsukura et al., 1998; Herold et al., 2006; Hallgren et al., 2007; Kim et al., 2008; Jones et al., 2009; Chan et al., 2010a; Chan et al., 2010b; Collington et al., 2010; Teijaro et al., 2011). Mice deficient in these cytokines or chemokines, which include IL-4, IFN- γ, CCL2, CCL3, and CCL5 (RANTES), have reduced but not complete absence of MCp recruitment to the lung (Hallgren et al., 2007; Jones et al., 2009; Collington et al., 2010). The expression of multiple, redundant molecules able to recruit MCps ensures that MCs arrive promptly at the site of infection and rapidly mount a defense against the virus, as supported by the lack of a significant phenotype in many singly-deficient mice.

## Pathways of Mast Cell Activation During Influenza A Virus Infection

MCs are activated during influenza infection through a variety of signals and stimuli present in the infected lung. Activation has been observed to occur through Fc-mediated signaling, PRRs, and cytokine and complement signaling (Reber et al., 2015). Activation of MCs early in infection likely occurs in response to stimulation by cytokines and other distress signals produced by infected cells and other immune and non-immune resident cells of the lung while stimulation during the adaptive immune response may include Fc-mediated activation (Graham et al., 2015; Marshall et al., 2019). Teasing these responses apart is difficult as multiple stimuli are likely interacting with MCs simultaneously (Gri et al., 2012). Furthermore, activating signals need not derive solely from other cells types, but can also be the result of autocrine and paracrine signaling from the MCs themselves (Oldford et al., 2018; Marshall et al., 2019).

As described in the previous section, infection of the epithelial-endothelial barrier and of alveolar macrophages releases a flood of chemoattractant molecules as well as a variety of activating and signaling cytokines, including type I IFNs, TNF-α, IL-1α, IL-1β, and IL-33 (Sanders et al., 2011; Teijaro et al., 2011). Engagement of these cytokines with their respective receptors on the surface of MCs triggers degranulation of the cell within minutes and initiates antiviral signaling cascades within the cell, leading to the transcription, translation and secretion of the MCs’ own repertoire of cytokines and chemokines. Cytokines such as IL-4, IL-5, IL-6, IL-8, IL-13, and TNF-α, chemokines such as CCL2 and CCL3, and the MC proteases tryptase and chymase can be detected in the supernatants of influenza infected MC cultures and in the bronchioalveolar lavage fluid (BALF) of both influenza patients and infected mice (**Table 2**) (Yu et al., 2011; Hu et al., 2012; Graham et al., 2013; Betakova et al., 2017). Multiple signals are required before MC degranulation occurs during respiratory infection. Infection of MCs in culture with rhinovirus or respiratory syncytial virus (RSV) or treatment with type I IFNs alone does not result in degranulation but does induce secretion of CCL4, CCL5, CXCL10, and additional type I and type III IFNs (Al-Afif et al., 2015; Akoto et al., 2017).

**Table 2:** *In vivo* cytokine and chemokine expression during influenza virus infection.

Human					
Strain		Tissue		Biological observations*	Reference
H1N1		Serum		Increased IL-6 expression correlates with disease severity	Chiaretti et al. (2013)
H1N1 (pandemic)		Plasma		Influenza patients had higher TNF-α, IFNγ, IL-2, IL-5, IL-6, IL-10, and IL-12 Severe influenza patients had increased IL-6 and IL-10 but lower TNF-α, IFNγ, and IL-5 compared to mild disease patients	Yu et al. (2011)
H7N9		Plasma		Increased TNF-α, IFNγ, IL-4, IL-10, and IL-12 in patients with severe disease	Han et al. (2014)
*Unknown*		Serum		Upregulation of IL-37 in influenza A patients	Zhou et al. (2019)
**Mouse**					
**Virus**	**Model**		**Tissue**	Biological observations*	**Reference**
H1N1	Balb/C		Lung	Differential cytokine and chemokine expression between lethal and sublethal infections	Turianova et al. (2019)
H1N1	Balb/C		Lung Serum	Young mice express less IL-10 and more IFNγ than adult mice, corresponding to increased immune pathology	Verhoeven and Perry (2017)
H1N1	C57BL/6 C57BL/6.IL-10^-/-^		BALF	IL-10^-/-^ mice had increased IFN-γ but reduced IFN-α expression	Sun et al. (2010)
H1N1	C57BL/6 C57BL/6.IL-10^-/-^		Lung	Elevated IL-6, IL-17, and IL-22 in IL-10^-/-^ mice	Mckinstry et al. (2009)
H1N1 H1N1 (pandemic)	C57BL/6 C57BL/6.IL-10^-/-^ C57BL/6.IL-10^-/-^		BALF	IL-6 signaling is essential for survival of infection and for prevention of virus-induced neutrophil cell death	Dienz et al. (2012)
H1N1	C57BL/6		BALF	SIPR signaling mediates induction of type I and II IFNs, IL-1 α, IL-6, TNF-α, CCL2, CCL3, CCL5, and CXCL2	Teijaro et al. (2011)
H1N1	C57BL/6 C57BL/6.IL-10^-/-^		Lung	IL-6 deficiency increases morbidity, TGF-β, and epithelial cell apoptosis IL-6 is essential for macrophage recruitment	Yang et al. (2017)
H1N1 (pandemic)	Balb/C		Lung	Increased transcription of inflammatory genes with mouse adaption of virus	Josset et al. (2012)
H1N1 (pandemic)	Balb/C		Lung BALF Serum	IL-37 treatment inhibited increase inflammatory cytokine expression	Qi et al. (2019)
H1N1 H5N1 H7N7 H7N9	Balb/C		Lung	Strain-dependent differential induction of type I and II IFNs, IL-6, CXCL10, and CCL4	Morrison et al. (2014)
H3N2	Balb/C		Skin	IAV antigen-IgE cutaneous anaphylaxis in IAV-recovered mice	Grunewald et al. (2002)
H3N2	C57BL/6 C57BL/6.TGFβ^-/-^		BALF	Reduced IFNβ, inflammation, and neutrophil, macrophage, and monocyte infiltration in TGFβ^-/-^ mice	Denney et al. (2018)
H5N1	C57BL/6		Lung	Upregulation of TNF-α, IFNγ, IL-1α, IL-6, IL-10, IL- 12, and chemokines Downregulation of IL-1β, IL-2, and IL- 9	Jang et al. (2012)
**Pig**					
**Virus**		**Tissue**		Biological observations*	**Reference**
H1N1		Lung		Higher mortality, lower TNF-α and IFNγ, and higher IL- 10 after alveolar macrophage depletion	Kim et al. (2008)

*Relative to controls.

MCs rely on activating signals in combination to dictate the appropriate induction and amounts of downstream mediators. For example, while IL-33 alone induces expression of the Intercellular Adhesion Molecule-1 (ICAM-1) on MCs, concomitant binding of neuropeptide Substance P to the cell surface strongly induces production of TNF (Drube et al., 2016; Taracanova et al., 2017). Similarly, IL-1 alone induces production of IL-3, IL-5, IL-6, IL-9, and TNF-α by MCs in human cultured MCs (HCMCs) and murine bone marrow-derived MCs (BMMCs) but in combination with IL-4, TNF- α, or a synthetic double-stranded RNA TLR3 agonist, IL-1 induces much higher levels of IL-5 and IL-6 secretion (Hultner et al., 2000; Kandere-Grzybowska et al., 2003; Kandere-Grzybowska et al., 2006; Nagarkar et al., 2012).

MCs express a wide variety of PRRs, capable of detecting an array of pathogen products. Intracellular pathogen detection and innate immune pathways play a role in MC activation in response to influenza virus infection (Sandig and Bulfone-Paus, 2012). The mouse mastocytoma cell line P815 has been used extensively to probe the cytokine and chemokine expression profiles of influenza-infected MCs. Expression of IL-6, IL-18, TNF-α, IFN-γ, and CCL2 increase within 24 h of infection of P815 cells with either a H1N1, H5N1, or H7N2 strain of IAV (Hu et al., 2012; Liu et al., 2014). Infection of P815 cells also causes significant cytopathic effect (CPE) and cell death, an effect not reported in influenza-infected MCs cultured from hematopoietic stem cells of the bone marrow (mice), peripheral blood (human), or umbilical cord blood (human) (Hu et al., 2012; Liu et al., 2014). Although cultured human and mouse MCs are vulnerable to infection by a number of strains of IAV, the infection appears to be largely abortive, producing little to no detectable progeny (Graham et al., 2013; Marcet et al., 2013; Ng et al., 2019). Nonetheless, replication of viral RNA and production of viral protein has been observed in cultured MCs (Kulka et al., 2004; Graham et al., 2013). In influenza inoculated BMMCs, signaling through double-stranded RNA-sensing PRRs RIG-I and TLR3 strongly induces IL-6 and the proinflammatory leukotriene LTB_4_ (Graham et al., 2013). Infection of cells lacking RIG-I or its adaptor MAVS fail to produce IL-6 or LTB_4_. The deficient cells, nonetheless, secreted equivalent levels of histamine to wild type cells, suggesting degranulation of MCs during influenza infection is independent of RIG-I signaling. Treatment of HCMCs and murine BMMCs with RSV, UV-inactivated influenza, or reovirus induces type 1 IFN secretion in a TLR3-dependent manner. BMMCs from *TLR3^-/-^* mice degranulate upon treatment the synthetic dsRNA PolyI:C but fail to induce type 1 IFNs (Kulka et al., 2004). PRR pathogen sensing function appears to function in parallel and in combination with cytokine, chemokine, and small molecule signaling to drive the MC response to infection.

Fc receptor-mediated stimulation of MCs likely occurs as the influenza infection progresses and B cells begin producing antibodies specific to the ongoing infection. IAV-specific IgE can be detected in mice one week after influenza infection, and intradermal rechallenge with IAV antigen results in MC degranulation and cutaneous anaphylaxis (Grunewald et al., 2002). The role of antiviral antibody responses in MC activation (*via* Fc receptor binding or complement proteins) during acute influenza infection has not been studied but serves to highlight the numerous and diverse stimuli presented to MCs over the course of infection. Not only do MCs encounter multiple stimuli at any given timepoint during infection, but the profile of the stimuli also evolves as additional innate cells arrive at the site of infection and later, as the immune response transitions from innate to adaptive. The vast assortment of activating receptors expressed by MCs allows the cells to detect fluctuations in the nature and volume of incoming signals and modulate their responses accordingly.

## Immunomodulatory Function of Mast Cells

The lungs of influenza virus infected patients contain a great number of cytokines, chemokines, and other immunomodulatory molecules (**Table 2**). Quantification of cytokine expression during human infection with various seasonal and pandemic strains of influenza shows differential expression not only across strains but between mild and severe disease as well. Patients with pandemic H1N1 2009, both mild (respiratory symptoms) and severe (pneumonia) cases, had elevated serum IL-10 and IL-6 compared to healthy controls and patients with mild or severe non-influenza illnesses (Yu et al., 2011). Severe cases had significantly higher IL-10 and IL-6 than mild cases, but no difference in IFN-γ, TNF-a, IL-1, IL-2, IL-4, IL-5, IL-6, IL-8, IL-10, IL-12, IL-17, or IL-23 was reported. Similar cytokine profiles have been recorded in patients infected with avian H5N1 and H7N2 strains (Betakova et al., 2017). Because severe influenza is linked to excessive inflammation in the lung, much effort has been expended in defining the role of these cytokines in mild and severe influenza.

While most studies examining the role of MCs during influenza virus infection have focused on MC-derived pro-inflammatory cytokines, a growing body of work highlights the immune suppressive or anti-inflammatory properties of MCs (Galli et al., 2008). The roles of individual pro-inflammatory cytokines and the cell types that produce them, including MCs, have been comprehensively reviewed elsewhere (Graham et al., 2015; Ramos and Fernandez-Sesma, 2015). Although the immune suppressive functions of MCs have not yet been examined in the context of influenza infection specifically, the effects have been identified in a broad array of immune insults, suggesting that immune suppression by MCs is a conserved function that contributes to the resolution of infection and tissue damage. In the following sections, we will discuss the potential contribution of MCs to immune suppression at the resolution of influenza infection.

### IL-10

Over the past decade, surprising evidence has emerged suggesting an important role of MC produced IL-10 in the regulation of inflammation during infection and tissue damage. In the skin, MCs play a vital role in reducing the pathology of allergic reactions such as to poison ivy and poison oak, as well as the sunburn caused by chronic exposure to ultraviolet B (UVB) light (Hart et al., 1998; Grimbaldeston et al., 2007). Mice lacking IL-10 and those lacking MCs altogether suffered significantly more tissue damage than wild type mice after exposure to poison oak or poison ivy or after prolonged exposure to UVB. In addition to increased tissue swelling and epithelial cell death at the site of exposure, IL-10-deficient and MC-deficient mice had higher infiltration of effector T cells and macrophages in the damaged tissue. Deficiency also delayed recovery and healing at the injured site. Of note, mice lacking IL-10 healed from UVB exposure more quickly than the MC-deficient mice, indicating additional anti-inflammatory mechanisms employed by MCs. The authors also found that IL-10 expression was reduced but not absent in mice with MCs lacking the common γ-chain of the Fc receptor and therefore, lacking signaling in response to IgE or IgG. The presence of intermediate levels of IL-10 expression in the absence of Fc-mediated signaling indicates that multiple signaling pathways induce IL-10 expression in MCs. Mosquito saliva elicits IL-10 production by MCs and reduces the inflammatory response at the bite (Depinay et al., 2006). Mice lacking MCs or IL-10 have increased tissue inflammation and higher levels of pro-inflammatory cytokines such as IFN-γ in response to mosquito saliva. Tissue damage caused by other animal venoms, such as those of reptiles and insects, is mitigated both by release of MC-derived proteases capable of degrading the venom and by suppression of aggressive inflammatory responses at the site of injury (Depinay et al., 2006; Metz et al., 2006; Schneider et al., 2007; Akahoshi et al., 2011; Marichal et al., 2013; Starkl et al., 2016). Interestingly, in models of mild contact hypersensitivity (CHS), MCs behave in a largely pro-inflammatory manner and express little to no IL-10 (Biedermann et al., 2000; Bryce et al., 2004; Norman et al., 2008; Dudeck et al., 2011; Reber et al., 2017). However, in severe CHS models, MCs appear to convert to an anti-inflammatory state, expressing high levels of IL-10 (Norman et al., 2008; Reber et al., 2017; Gaudenzio et al., 2018; Galli et al., 2020). Although not directly correlative to the differential disease severity caused by different IAV strains, this work suggests that the anti-inflammatory functions of MCs may be particularly responsive when the inflammatory state is severe.

Although IL-10 is consistently upregulated in severe influenza cases in humans, the full extent of the immune regulatory role of IL-10 during influenza infection has not been explored. A case study of a child with a deletion in the *IL10RA* gene resulted in excessive pro-inflammatory cytokine expression and fatal encephalitis as a result of influenza infection (Ishige et al., 2018). His death is attributed to the failure of IL-10 to mitigate the influenza-induced inflammatory response, suggesting that IL-10 is vital to tempering severe influenza-mediated inflammation. Studies examining the role of IL-10 in the immune response to influenza infection have produced conflicting results, however. Similar to the strong induction of IL-10 in patients with severe influenza, infection of Balb/C mice with a lethal dose (LD100) of H1N1 A/Puerto Rico/8/34 (PR8) induced strong IL-10 expression (Turianova et al., 2019). A lower, nonlethal dose (LD0) did not induce IL-10. Furthermore, *Il10^-/-^* Balb/C mice infected with a nonlethal dose of H1N1 PR8 show identical morbidity to wild type Balb/C mice (Mckinstry et al., 2009). When challenged with a lethal dose of H1N1 PR8, the IL-10-deficient mice had reduced mortality but equivalent weight loss and only slightly elevated inflammation when compared their wild type counterparts at days 6-7 post-infection. A study by Sun, et al. likewise observed similar lung pathology and immune cell infiltration in IL-10-deficient and IL-10-sufficient C57BL/6 mice infected with nonlethal and lethal doses of H1N1 PR8 (Sun et al., 2010). In contrast, several studies have shown that absence of IL-10 signaling results in enhanced lung pathology, partially attributed to high levels of CD8+ T cells activity in mice (Zou et al., 2014). Antibody blockade of the IL-10 receptor severely reduced survival of Balb/C mice infected with H1N1 PR8 (Sun et al., 2009). *In vitro*, addition of IL-10 to cultures of splenic CD8+ T cells collected from mice 5 days post-influenza infection halted proliferation of these cells (Zou et al., 2014). Dutta, et al. proposes a biphasic effect of IL-10 during infection wherein IL-10 released early in infection inhibits viral neuraminidase enzymatic function (Dutta et al., 2015). As infection wanes and the concentration of viral particles decreases, the IL-10 is available to bind to IL-10R and activate negative immune regulation. Injection of IL-10-deficient mice with exogenous IL-10 at 4 days post-infection (p.i.) greatly reduced morbidity, and all IL-10 recipient mice survived infection. Young mice, roughly equivalent to human infants and toddlers, produce less IL-10 in the lungs and at a later time point than adult mice during influenza infection (Verhoeven and Perry, 2017). During infection, young mice also have higher levels of macrophage infiltration, immune pathology, delayed viral clearance, and delayed antibody production, suggesting the lower levels of IL-10 delay the switch from innate to adaptive responses. The reduced IL-10 response in young children may partially explain why they are among the most vulnerable to developing severe influenza. The role of IL-10 in mitigating influenza infection appears to depend on a number of factors, not only the age of the patient, but the strain and dosage of the infecting virus as well. Additional work is needed to understand if these conflicting data are reproducible in more outbred animal models or humans, and whether the use of strains other than H1N1 PR8, a laboratory strain passaged outside of humans for more than 70 years, will yield different and perhaps more interpretable results.

### IL-6

IL-6 has been implicated as a master controller of the shift from the innate to the adaptive immune response (Xing et al., 1998; Hurst et al., 2001; Jones, 2005). A human polymorphism in the promoter region of the human *IL6* gene contributes to increased IL-6 production. People with the -174G polymorphism have lower incidence of acute respiratory distress syndrome (ARDS) and mortality associated with ARDS in cases of community-acquired pneumonia (Terry et al., 2000; Martin-Loeches et al., 2012). People with an *IL6* polymorphism resulting in low IL-6 production have increased susceptibility to disease after rhinovirus or RSV exposure (Fishman et al., 1998). In agreement with these findings, commonly prescribed drugs that target IL-6 or its receptor IL-6R for treating inflammatory diseases such as rheumatoid arthritis also increase risk of both upper and lower respiratory infection (Lang et al., 2012; Van Rhee et al., 2014). Finally, in a murine acute lung injury and ARDS model, MC-derived IL-6 induces apoptosis in lung-infiltrating neutrophils, which are also known to contribute to influenza-associated inflammation and immune pathology (Ganeshan et al., 2013).

Like IL-10, IL-6 is highly upregulated during severe IAV infection of both humans and mice (Yu et al., 2011; Chiaretti et al., 2013; Betakova et al., 2017). While the high levels of IL-6 in severe influenza have been taken as an indication that IL-6 plays a role in exacerbating lung inflammation, recent work has demonstrated that IL-6 is, in fact, essential for reducing lung pathology during influenza infection. IL-6-deficient mice have increased morbidity and mortality when infected with H1N1 A/WSN/33 (Yang et al., 2017). When infected with H1N1 PR8 or pandemic H1N1 A/California/7/2009 at a dose that is sublethal in wild type mice, all IL-6-deficient mice died between 10 and 12 days p.i. (Dienz et al., 2012; Montier et al., 2012). Wild type and IL-6 deficient mice had equal numbers of inflammatory cells present in the lungs on days 9 through 11 p.i. However, both IL-6-deficient and IL-6R-deficient mice had extensive vascular leakage, emphysema-like swelling of the alveoli, and widespread cell death of the airway epithelium (Dienz et al., 2012; Yang et al., 2017). IL-6 deficient mice also had increased collagen deposition and fibroblast accumulation, indicative of developing fibrosis (Yang et al., 2017). In culture, IL-6-mediated crosstalk between human intestinal MCs and fibroblasts promotes MC survival, while also inducing fibroblast apoptosis through the production of the fibrolytic enzyme matrix metalloproteinase 1 (MMP-1) (Montier et al., 2012). IL-6-deficient mice did not develop severe lung pathologies until late in the course of infection, at a time when wild type mice are typically recovering, suggesting that the absence of IL-6 compromises recovery of the lung after infection.

An examination of the anti-inflammatory effects of IL-6 revealed increased levels of TNF-α, IFN-γ, GM-CSF, and CXCL2 in IL-6 deficient mice after inhalation or intraperitoneal injection of endotoxin (Xing et al., 1998). The survival rate of endotoxemic IL-6 deficient mice was 50% lower than wild type mice injected with endotoxin. Exogenous administration of IL-6 reduced pro-inflammatory cytokine expression to wild type level. Reciprocal regulation of IL-6 and TNF-α has been well established (Petersen and Pedersen, 2006; Tanaka et al., 2014). While TNF-α-induced NFĸB signaling induces IL-6 production, IL-6 appears to inhibit TNF-α expression, through upregulation of microRNAs that prevent TNF-α mRNA translation, downregulation of TNF-α-inducing PRRs, and induction of other anti-inflammatory cytokines such as IL-10 (Aderka et al., 1989; Schindler et al., 1990; Tanaka et al., 2014; Li et al., 2015). Administration of a TNF-α-blocking antibody rescues wild type mice from lethal endotoxin exposure (Barton and Jackson, 1993). Similarly, antibody inhibition of TNF-α reduces lung pathology, levels of IFN-γ, IL-4, and IL-5, and weight loss in mice infected with influenza or RSV (Hussell et al., 2001). The absence of TNF-α did not impair viral clearance of either infection. IL-6 and IL-13 produced by MCs stimulated with IL-33 drives polarization of anti-inflammatory alternatively activated (M2) macrophages, and in a murine model of autoimmune encephalitis, inhibited pro-inflammatory cytokine secretion by T cells (Finlay et al., 2020). In spite of the pathological role of IL-6 and TNF-α in chronic inflammatory diseases, the anti-inflammatory contribution of IL-6 to the resolution of infectious disease through mitigation of damage to the lung architecture, and reduction of proinflammatory cytokine production is clear (Tanaka et al., 2018). The role of MCs in mediating IL-6 expression during influenza infection, however, remains to be fully explored.

### Additional Cytokines With Immune Suppressive Potential

The complex and rapidly changing immune landscape during infection presents a great challenge to fully mapping the signaling networks and downstream effects of MCs and the cytokines these cells produce. In addition to IL-10 and IL-6, MCs produce an array of cytokines with both pro- and anti-inflammatory functions (Mukai et al., 2018; Galli et al., 2020). MC-derived cytokines of potential interest in suppressing influenza immunopathology include: TNFα, TGF-β1, GM-CSF, IL-2, IL-9, IL-13, and IL-33 (De Vries and Noelle, 2010; Mukai et al., 2018). However, these cytokines are also expressed by a number of other immune cell types during infection, and the cell type of origin appears to play a significant role in the downstream signaling, likely due to co-expression of other cytokines and the specific microenvironment.

For example, autocrine and paracrine signaling by MC-derived TGF-β1 restricts the release of a variety of mediators, including IL-6, IL-13, TNF, GM-CSF, and histamine (Gordon and Galli, 1994; Bissonnette et al., 1997; Lindstedt et al., 2001; Zhao et al., 2008; Fernando et al., 2013). In contrast, membrane-bound TGF-β1 on regulatory T cells (T_regs_) induces IL-6 production by MCs (Ganeshan and Bryce, 2012). Like TGF-β1, the outcome of TNFα expression appears heavily dependent on cell source, tissue localization, and TNF receptor (TNFR) expression (De Vries and Noelle, 2010). One of the earliest sources of TNFα during infection, MCs can release pre-formed TNFα *via* degranulation immediately upon pathogen detection (Gordon and Galli, 1990). Immunopathology attributable to excessive TNFα has been observed in a number of infections as well as inflammatory disorders (Bradley, 2008). Treatment of mice with an TNF-a inhibitor at the time of lethal H1N1 influenza infection conferred a protective advantage over untreated mice (Shi et al., 2013). Interestingly, this protective effect was not observed in mice infected with an H5N1 strain of influenza (Salomon et al., 2007). TNFα-deficient mice infected with a sublethal dose, however, presented with greater lung pathology, inflammatory cell infiltration, and recovered from infection more slowly than wild type mice (Damjanovic et al., 2011). These studies suggest a biphasic role for TNFα in the immune response to influenza infection, similar to IL-6 described in the previous section.

IL-9 has similar paradoxical effects on the inflammatory environment. Produced by T helper Th9 cells as well as MCs and type 2 innate lymphoid cells (ILC2s), IL-9 is strongly upregulated in patients infected with avian H7N9 influenza and in mice infected with H1N1 or H5N1 (Hultner et al., 2000; Stassen et al., 2000; Jang et al., 2012; Hamada et al., 2013; Han et al., 2014; Artis and Spits, 2015; Kaplan et al., 2015). Autocrine IL-9 and Th9-derived IL-9 contribute not only to MC recruitment and proliferation in the lung but also to TGF-β1 production by MCs, which can have suppressive effects on MCs as well as other immune cells as discussed above (Hultner et al., 1990; Godfraind et al., 1998; Matsuzawa et al., 2003; Kearley et al., 2011; Sehra et al., 2015). Like TGF-β1, IL-9 expression and function appears to be regulated in a location- and temporally-specific manner.

Likewise, release of GM-CSF by MCs may play a role both in increasing inflammation and in mitigating the development of immune pathology and lung injury. Responsible for immune cell maturation, recruitment, and activation, GM-CSF in high levels is a source of inflammation in multiple sclerosis, arthritis, Kawasaki disease, chronic obstructive pulmonary disease, and lung interstitial disease (Becher et al., 2016; Hamilton, 2019). During influenza infection, however, GM-CSF appears to play an important role in reducing inflammation and inducing anti-inflammatory M2 macrophages (Huang et al., 2011; Rosler and Herold, 2016). Mice lacking GM-CSF succumbed to lethal H1N1 and H3N2 infections more quickly than wild type mice, while all GM-CSF-overexpressing transgenic mice survived infection, had lower viral titers, and reduced lung pathology (Huang et al., 2011; Subramaniam et al., 2015). Intranasal administration of recombinant GM-CSF protected GM-CSF-deficient mice from lethal infection (Huang et al., 2011). GM-CSF overexpression reduced inflammatory cytokine levels and drove macrophage polarization toward the immune suppressive M2 phenotype (Halstead et al., 2018). While the role of MC-derived GM-CSF during influenza has yet to be examined, MC-deficient mice reconstituted with GM-CSF^-/-^ MCs reject skin allografts. In contrast, GM-CSF sufficient mice tolerated engraftment through recruitment long-lived, immune-suppressing dendritic cells, suggesting that through GM-CSF, MCs direct localization and function of additional immune regulatory cell types.

The contribution of individual cytokines to the defense against influenza infection and to what degree their activity drives or impedes the inflammatory tissue damage associated with severe disease will require extensive further research. Differentiating between the positive and negative outcomes of the signaling of a single cell type or a single cytokine, however, is severely impeded by the complex networks of cell signaling in the inflammatory environment, particularly over the course of viral infection. To fully understand the impact of these cytokines in the complex immune environment of an ongoing infection, the many cell types and the mediators they produce will have to be examined both individually and as a part of the whole. While a daunting endeavor, advances in mouse genetics, live imaging, and immune phenotyping have greatly expanded the toolset for examining such complex networks (Reber et al., 2017; Galli et al., 2020).

### Other Mast-Cell Derived Immune Mediators

MCs modulate the immune environment not only through the production of cytokines and chemokines but also by the expression of molecules capable of inhibiting or degrading cytokines and other signaling molecules produced by other cells. For example, MCs purified from human lungs respond to IgE-stimulation by producing the IL-1 receptor antagonist (IL-1Ra) (Hagaman et al., 2001). IL-1Ra binds the IL-1 receptor (IL-1R) with high affinity, outcompeting IL-1α and IL-1β and preventing the pro-inflammatory signaling of these cytokines (Dinarello, 2019). Production of the glucosaminoglycan heparin by MCs reduces inflammatory cell migration by downregulating intercellular adhesion molecule-1 (ICAM-1) on endothelial cells and blocking selectin and chemokine receptor binding of circulating leukocytes (Nelson et al., 1993; Miller et al., 1998; Kuschert et al., 1999; Wang et al., 2002). In addition to halting the migration of immune cells into the target tissue, heparin downregulates proinflammatory cytokine expression by downregulating ERK and NF-ĸB signaling in lung epithelial cells (Yi et al., 2015). MC proteases also have anti-inflammatory effects, degrading proinflammatory cytokines and alarmins such as TNF and IL-33 (Piliponsky et al., 2012; Hendrix et al., 2013; Roy et al., 2014). Theoharides, et al. has proposed a mechanism for MC-mediated control of the anti-inflammatory cytokine IL-37 through alternating expression of heparin and tryptase (Theoharides et al., 2019). IL-37 producing cells, such as macrophages, NK cells, activated B cells, and epithelial cells, secrete both the highly active IL-37 and the moderately active pro-IL-37, which requires protease cleavage to be fully active (Eisenmesser et al., 2019; Li et al., 2019). Heparin promotes formation of an inactive homodimer of IL-37 while protease cleavage converts pro-IL-37 to the fully active form, similar to the cleavage required to produce mature IL-33 (Lefrancais et al., 2014; Cavalli and Dinarello, 2018; Eisenmesser et al., 2019). Theoharides, et al. speculate that MCs may modulate the degree of inflammation at the site of infection by temporal modulation of heparin-mediated IL-37 inhibition and tryptase-mediated IL-37 activation (Theoharides et al., 2019). This hypothesis is particularly intriguing in regard to influenza infection: although IL-37 is detectable in the humans and mice throughout influenza infection, exogenous administration of IL-37 only produced beneficial effects in infected mice late in infection, suggesting that IL-37 may be sequestered in some manner early in infection (Qi et al., 2019; Zhou et al., 2019). Influenza infection induces a variety of cytokines in the lung; how MC mediators that activate, inhibit, or degrade those cytokines control the pro- or anti-inflammatory environment in the lung remains to be fully investigated.

## Mast Cells in Other Viral Infections

Whether MCs have a net positive or negative effect on influenza infection has yet to be determined and likely depends largely on the severity of infection. Human MC deficiency has never been identified, though as a result of being clinically inconspicuous or lethally critical for embryonic development is unknown (Rodewald and Feyerabend, 2012). Mastocytosis—excessive proliferation and accumulation of MCs—appears to rarely cause pulmonary symptoms, though rhinitis is common (Castells and Austen, 2002). Systemic mastocytosis patients participating in a clinical trial of the Kit tyrosine kinase inhibitor midostaurin, however, reported increased upper respiratory tract infections during treatment, leading a number to discontinue the trial (Kasamon et al., 2018). Midostaurin, and other tyrosine kinase inhibitors used to treat mastocytosis such as imatinib, are not Kit-specific inhibitors (Karaman et al., 2008). In addition to Kit, midostuarin binds tyrosine kinases FLT3, VEGFR, and PDGFR, inhibiting maturation and activation of a number of immune cell types, including NK cells and DCs (Huang et al., 2010; Wiernik, 2010; Valent et al., 2017; Gutierrez et al., 2018). Whether the increased risk of respiratory infection in mastocytosis-patients treated with midostaurin is the result of reduced mast cell numbers in combination with the inhibition of other immune cell types has not yet been investigated (Valent et al., 2017).

*In vivo* studies of MC are most commonly conducted in mice deficient in MCs as a results of mutations in *c-kit*, although these mice have non-MC hematopoietic abnormalities that have prompted the development of *Kit*-independent MC-deficient mice (Galli et al., 2020). Influenza infection of MC-deficient *Kit* mice has produced contradicting results. When infected with H1N1 A/WSN/33, MC-deficient mice exhibited reduced morbidity and mortality compared to MC-sufficient C57BL/6 mice (Graham et al., 2013). MC-deficient *Kit* mice adoptively transferred with murine BMMCs also had elevated morbidity and mortality, on par with the C57BL/6 mice. The lungs of infected C57BL/6 mice had higher levels of macrophage and neutrophil infiltration and TNF-α than the infected MC-deficient mice. In contrast, after infection with H1N1 PR8, no differences in morbidity or mortality were observed regardless of MC sufficiency. Clearly, strains of even the same influenza subtype induce disparate immune responses. Examination of the immune profiles, including cell types and cytokines, over the duration of infection with different influenza strain and subtypes, both human isolates and mouse-adapted strains, will be required to unravel the different responses induced by different viruses.

Studies of other viral infections provide insight into what immunomodulatory roles MCs may be playing during influenza infection (**Table 3**). In a murine model of vaccinia virus infection, dermal inoculation of the virus produces a large skin lesion in MC-deficient mice within 24 h that continues to expand over time (Domenico et al., 2012; Wang et al., 2012a; Wang et al., 2012b). In contrast, MC-sufficient mice and MC-reconstituted mice develop only small pinpoint lesions 3 days post-infection. MC-deficient mice had much higher viral loads at the site of infection as well as in the spleen compared to MC sufficient mice (Wang et al., 2012a). MC numbers in MC-sufficient and MC-reconstituted mice rose significantly at the site of lesions, although whether the increase was a result of recruitment of MC progenitors or proliferation of resident MCs was not investigated. High expression of both TNF-α and IL-6 were present in the MC-deficient lesions, suggesting an anti-inflammatory role for MCs in vaccinia infection. Of particular interest was the expression of IL-6 which is not usually seen in vaccinia infection, suggesting MCs may control pro-inflammatory IL-6 expression by other cell types. In addition to anti-inflammatory responses by MCs, the authors observed vaccinia-induced degranulation of MCs that contributed to viral clearance.

**Table 3:** *In vivo* studies of mast cells during viral infections.

Human				
Virus/strain	Tissue		Biological observations*	Reference
IAV RSV adenovirus	Lung		Recruitment of mast cell progenitors to the lungs	Andersson et al. (2018)
**Mouse**				
**Virus/strain**	**Model**	**Tissue**	Biological observations*	**Reference**
H1N1	Balb/C	Lung	Innate immunity-mediated recruitment and proliferation of mast cell progenitors in the lung	Zarnegar et al. (2017) Zarnegar et al. (2018)
H1N1	C57BL/6 B6.Cg-Kit^w-sh^	BALF	Reduced inflammation and mortality in mast cell-deficient mice infected with A/WSN/33 but not PR8	Graham et al., 2013
H1N1	C57BL/ 10.D2 B10.D2 IL-10^-/-^ B10.D2.6.5 IL-10^-/-^	Lung	Proposes biphasic activity of IL-10: (1) inhibition virus-mediated activation of TGF-β early in infection; (2) suppression of inflammation late in infection	Dutta et al. (2015)
H5N1	Balb/C	Lung	Ketotifen reduces mast cell release of histamine, tryptase, and TNF-α	Hu et al. (2012)
H5N1	Balb/C	Lung Nose Trachea	Sodium cromoglycate reduce mast cell-induced inflammatory responses	Han et al. (2016)
Western reserve vaccinia	Kit^+/+^ Kit^W-sh^	Skin	Mast cell deficiency results in severe ulcerated skin lesions and high levels of inflammatory cell infiltration	Domenico et al. (2012)
Western reserve vaccinia	Kit^+/+^ Kit^W-sh^	Skin Spleen	TLR2-mediated degranulation of mast cells	Wang et al. (2012a) Wang et al. (2012b)
HSV-1	Kit^+/+^ Kit^W-sh^	Eye	Mast cell deficiency results in elevated neutrophil infiltration, neutrophil infection and dissemination of virus	Royer et al. (2015)
HSV-2	Kit^+/+^ Kit^W-v^	Skin	Mast cell derived-TNF-α and -IL-6 protect mice from HSV mortality	Aoki et al. (2013)
DENV1 DENV2 DENV3 DENV4	Kit^+/+^ Kit^W-sh^	Skin Draining lymph nodes	Mast cell-mediated recruitment of recruit NK cells	St John et al. (2011)
DENV2	Kit^+/+^ Kit^W-sh^	Ear Spleen Peritoneal/mesentary tissue	Mast cells contribute to vascular permeability through chymase and leukotriene secretion	St John et al. (2013)
DENV2	Kit^+/+^ Kit^W-sh^	Skin	Mast cells reduce CCL2-mediated infiltration of inflammatory macrophages	Chu et al. (2015)
DENV1 DENV2	C57BL/6 Kit^W-sh^ Fcgr3-/-	Peritoneal tissue Liver	Mast cells reduce CCL2-mediated infiltration of inflammatory macrophages	Syenina et al. (2015)

*Relative to controls.

In contrast to vaccinia infection, the presence of IL-6 and TNF-α produced by MCs was found to be essential for minimizing inflammation and viral replication in a murine skin model of herpes simplex-2 (HSV-2) infection (Aoki et al., 2013). Reconstitution of MC-deficient mice with *IL-6^-/-^* or *TNF-α^-/-^* MCs failed to reduce HSV-2 morbidity and mortality. In an ocular model of herpes simplex-1, MC deficiency also led to increased viral replication and inflammation as a result of increased neutrophil infiltration (Royer et al., 2015). In addition, HSV-2-infected neutrophils from the cornea facilitated dissemination of the virus to the nervous system.

As with influenza, the study of MCs during dengue virus infection has largely concentrated on the negative effects of MC activation and signaling. MCs have been implicated in the vascular leakage associated with severe dengue diseases. Activated MCs restructure the neighboring vasculature to facilitate recruitment of circulating immune cells; excessive release of chymase and tryptase, as found in the plasma of severe dengue patients, may contribute to systemic vascular permeability and hypovolemia (St John et al., 2013). As severe dengue and dengue hemorrhagic fever are most common during heterotypic secondary dengue infection, the production of heterotypic IgE may contribute to over-activation of MCs (Syenina et al., 2015). However, in the absence of MCs, dengue-infected mice exhibited increased inflammation at the site of infection and higher numbers of infiltrating inflammatory cells such as macrophages (Chu et al., 2015). MC-deficient mice also had increased viral burden and bleeding tendency, indicating failure to fully activate platelets and vascular endothelial cells in response to injury (Chen et al., 2009; Chu et al., 2015). At the site of infection, MCs in the skin produce chemokines to recruit NK and NKT cells to the skin and prevent spread of the virus to the draining lymph nodes (St John et al., 2011). Like influenza, MCs appear to play an important role activating and administrating the immune response at the site of dengue infection, and again like influenza, the net positive or negative outcome of MC activation appears to be highly context dependent.

## Mast cells and Mast Cell Mediators as Therapeutic Targets for Preventing Immune Pathology

As has been discussed above, MCs and the mediators they produce are plastic and vary significantly over the duration of an infection. As described above, exogenous expression of IL-10, IL-37, or GM-CSF in the lungs of influenza-infected mice improved survival and recovery from infection (Huang et al., 2011; Dutta et al., 2015; Subramaniam et al., 2015; Qi et al., 2019). These studies suggest great potential in the use of MC-derived mediators as therapeutics for immune pathology and protection against an excessive and damaging inflammatory response. Therapeutically targeting MCs or their mediators will need careful consideration in order to prevent off-target effects, however. Blocking the anti-inflammatory functions or enhancing the pro-inflammatory functions may result in excessive tissue damage resulting from infiltration and activation of cytotoxic cells. Treatment of mice with an FDA-approved TNFα inhibitor increased survival but prolonged and exacerbated lung pathology, raising concerns for the risk of severe influenza in patients prescribed TNFα-blocking drugs commonly used to treat rheumatoid arthritis, irritable bowel syndrome, and psoriasis (Shale et al., 2010; Damjanovic et al., 2011; Shi et al., 2013).

Identifying suitable MC mediators for therapeutic targeting will take careful consideration in order not to attenuate the beneficial role MCs play in both inducing and suppressing inflammation and the immune response. Use of mast cell stabilizing drugs such as sodium cromoglycate and ketotifen, which prevent mast cell degranulation and histamine release, as well as inhibitors of receptors that bind MC products such as leukotrienes have shown promise in animal models of influenza and dengue virus infection (Hu et al., 2012; St John et al., 2013; Han et al., 2016). Mast cell stabilizers, alone or in combination with the influenza antiviral oseltamivir, improved mice survival after H5N1 influenza challenge (Hu et al., 2012; Han et al., 2016). In a murine model of severe dengue, treatment mast cell stabilizers or the leukotriene receptor antagonist montelukast reduced vascular permeability and pathologic vascular leakage in infected mice (St John et al., 2013). These studies demonstrate that viability of targeting therapeutics to diminish specific inflammatory responses of mast cells, such as degranulation, without blunting the anti-inflammatory function of the MCs. To utilize the immunomodulatory effect of MCs to combat infection will require a better understanding of the various outcomes of MC mediator expression and signaling. Further, significantly more work is required to better understand the effects of over the counter or prescription pharmacological agents targeting MC functions, such as histamine release, in regulating infection and tissue damage.

## Concluding Remarks

MCs play a far-reaching role in immune responses, stimulating the pro-inflammatory environment and recruitment of addition immune responders, as well as driving immune suppression and reduction of the inflammatory environment. This role for MCs in the context of infection is under-studied, but as many infections, such as influenza, cause tissue damage as a result of inflammation, a better understanding of the role of MCs in mitigating this damage may provide insight into better management of immunopathology during infection. Although no studies have yet identified an immune suppressive role for MCs in resolving influenza infection, immune mediators such as IL-10 and IL-6 are known to play an important role in mitigating influenza pathology. Many cell types produce immune suppressive cytokines, and identifying individual sources and roles for each is difficult. However, the contribution of each cell type present at the site of infection cannot be discounted, and a better understanding of the regulatory networks driving the immune responses to influenza is essential to developing new and more effective treatments.

## Author Contributions

AM-S and SP conceptualized the content of and wrote the article. All authors contributed to the article and approved the submitted version.

## Funding

This work was supported by NIH RO1AI130065 (SP), T32AI007244 (AM-S), and unrestricted funds from The Scripps Research Institute, La Jolla, CA (SP).

## Conflict of Interest

The authors declare that the research was conducted in the absence of any commercial or financial relationships that could be construed as a potential conflict of interest.

## References

[B1] Abonia J. P. Hallgren J. Jones T. Shi T. Xu Y. Koni P.. (2006). Alpha-4 integrins and VCAM-1, but not MAdCAM-1, are essential for recruitment of mast cell progenitors to the inflamed lung. Blood 108, 1588–1594. 10.1182/blood-2005-12-012781 16670268 PMC1895513

[B2] Abraham S. N. Malaviya R. (1997). Mast cells in infection and immunity. Infect. Immun. 65, 3501–3508. 10.1128/IAI.65.9.3501-3508.1997 9284112 PMC175499

[B3] Abraham S. N. St John A. L. (2010). Mast cell-orchestrated immunity to pathogens. Nat. Rev. Immunol. 10, 440–452. 10.1038/nri2782 20498670 PMC4469150

[B4] Aderka D. Le J. M. Vilcek J. (1989). IL-6 inhibits lipopolysaccharide-induced tumor necrosis factor production in cultured human monocytes, U937 cells, and in mice. J. Immunol. 143, 3517–3523. 10.1159/000417355 2584704

[B5] Adlhoch C. Gomes Dias J. Bonmarin I. Hubert B. Larrauri A. Oliva Dominguez J. A.. (2019). Determinants of Fatal Outcome in Patients Admitted to Intensive Care Units With Influenza, European Union 2009-2017. Open Forum Infect. Dis. 6, ofz462. 10.1093/ofid/ofz462 32258201 PMC7105050

[B6] Agier J. Zelechowska P. Kozlowska E. Brzezinska-Blaszczyk E. (2016). Expression of surface and intracellular Toll-like receptors by mature mast cells. Cent. Eur. J. Immunol. 41, 333–338. 10.5114/ceji.2016.65131 28450795 PMC5382879

[B7] Akahoshi M. Song C. H. Piliponsky A. M. Metz M. Guzzetta A. Abrink M.. (2011). Mast cell chymase reduces the toxicity of Gila monster venom, scorpion venom, and vasoactive intestinal polypeptide in mice. J. Clin. Invest. 121, 4180–4191. 10.1172/JCI46139 21926462 PMC3195461

[B8] Akoto C. Davies D. E. Swindle E. J. (2017). Mast cells are permissive for rhinovirus replication: potential implications for asthma exacerbations. Clin. Exp. Allergy 47, 351–360. 10.1111/cea.12879 28008678 PMC5396281

[B9] Akula S. Paivandy A. Fu Z. Thorpe M. Pejler G. Hellman L. (2020). Quantitative In-Depth Analysis of the Mouse Mast Cell Transcriptome Reveals Organ-Specific Mast Cell Heterogeneity. Cells 9. 10.3390/cells9010211 PMC7016716 31947690

[B10] Al-Afif A. Alyazidi R. Oldford S. A. Huang Y. Y. King C. A. Marr N.. (2015). Respiratory syncytial virus infection of primary human mast cells induces the selective production of type I interferons, CXCL10, and CCL4. J. Allergy Clin. Immunol. 136, 1346–1354 e1341. 10.1016/j.jaci.2015.01.042 25819983

[B11] Andersson C. K. Shikhagaie M. Mori M. Al-Garawi A. Reed J. L. Humbles A. A.. (2018). Distal respiratory tract viral infections in young children trigger a marked increase in alveolar mast cells. ERJ Open Res. 4, 00038–2018. 10.1183/23120541.00038-2018 30480000 PMC6250563

[B12] Aoki R. Kawamura T. Goshima F. Ogawa Y. Nakae S. Nakao A.. (2013). Mast cells play a key role in host defense against herpes simplex virus infection through TNF-alpha and IL-6 production. J. Invest. Dermatol. 133, 2170–2179. 10.1038/jid.2013.150 23528820

[B13] Arock M. Ross E. Lai-Kuen R. Averlant G. Gao Z. Abraham S. N. (1998). Phagocytic and tumor necrosis factor alpha response of human mast cells following exposure to gram-negative and gram-positive bacteria. Infect. Immun. 66, 6030–6034. 10.1128/IAI.66.12.6030-6034.1998 9826392 PMC108768

[B14] Artis D. Spits H. (2015). The biology of innate lymphoid cells. Nature 517, 293–301. 10.1038/nature14189 25592534

[B15] Atkin-Smith G. K. Duan M. Chen W. Poon I. K. H. (2018). The induction and consequences of Influenza A virus-induced cell death. Cell Death Dis. 9, 1002. 10.1038/s41419-018-1035-6 30254192 PMC6156503

[B16] Baram D. Vaday G. G. Salamon P. Drucker I. Hershkoviz R. Mekori Y. A. (2001). Human mast cells release metalloproteinase-9 on contact with activated T cells: juxtacrine regulation by TNF-alpha. J. Immunol. 167, 4008–4016. 10.4049/jimmunol.167.7.4008 11564820

[B17] Barton B. E. Jackson J. V. (1993). Protective role of interleukin 6 in the lipopolysaccharide-galactosamine septic shock model. Infect. Immun. 61, 1496–1499. 10.1128/IAI.61.4.1496-1499.1993 8454355 PMC281391

[B18] Becher B. Tugues S. Greter M. (2016). GM-CSF: From Growth Factor to Central Mediator of Tissue Inflammation. Immunity 45, 963–973. 10.1016/j.immuni.2016.10.026 27851925

[B19] Betakova T. Kostrabova A. Lachova V. Turianova L. (2017). Cytokines Induced During Influenza Virus Infection. Curr. Pharm. Des. 23, 2616–2622. 10.2174/1381612823666170316123736 28302021

[B20] Biedermann T. Kneilling M. Mailhammer R. Maier K. Sander C. A. Kollias G.. (2000). Mast cells control neutrophil recruitment during T cell-mediated delayed-type hypersensitivity reactions through tumor necrosis factor and macrophage inflammatory protein 2. J. Exp. Med. 192, 1441–1452. 10.1084/jem.192.10.1441 11085746 PMC2193186

[B21] Bissonnette E. Y. Enciso J. A. Befus A. D. (1997). TGF-beta1 inhibits the release of histamine and tumor necrosis factor-alpha from mast cells through an autocrine pathway. Am. J. Respir. Cell Mol. Biol. 16, 275–282. 10.1165/ajrcmb.16.3.9070612 9070612

[B22] Bradley J. R. (2008). TNF-mediated inflammatory disease. J. Pathol. 214, 149–160. 10.1002/path.2287 18161752

[B23] Bryce P. J. Miller M. L. Miyajima I. Tsai M. Galli S. J. Oettgen H. C. (2004). Immune sensitization in the skin is enhanced by antigen-independent effects of IgE. Immunity 20, 381–392. 10.1016/S1074-7613(04)00080-9 15084268

[B24] Castells M. Austen K. F. (2002). Mastocytosis: mediator-related signs and symptoms. Int. Arch. Allergy Immunol. 127, 147–152. 10.1159/000048188 11919427

[B25] Caughey G. H. (2011). Mast cell proteases as protective and inflammatory mediators. Adv. Exp. Med. Biol. 716, 212–234. 10.1007/978-1-4419-9533-9_12 21713659 PMC3954859

[B26] Cavalli G. Dinarello C. A. (2018). Suppression of inflammation and acquired immunity by IL-37. Immunol. Rev. 281, 179–190. 10.1111/imr.12605 29247987

[B27] Chan M. C. Cheung C. Y. Chui W. H. Tsao S. W. Nicholls J. M. Chan Y. O.. (2005). Proinflammatory cytokine responses induced by influenza A (H5N1) viruses in primary human alveolar and bronchial epithelial cells. Respir. Res. 6, 135. 10.1186/1465-9921-6-135 16283933 PMC1318487

[B28] Chan M. C. Chan R. W. Yu W. C. Ho C. C. Yuen K. M. Fong J. H.. (2010a). Tropism and innate host responses of the 2009 pandemic H1N1 influenza virus in ex vivo and in vitro cultures of human conjunctiva and respiratory tract. Am. J. Pathol. 176, 1828–1840. 10.2353/ajpath.2010.091087 20110407 PMC2843473

[B29] Chan R. W. Yuen K. M. Yu W. C. Ho C. C. Nicholls J. M. Peiris J. S.. (2010b). Influenza H5N1 and H1N1 virus replication and innate immune responses in bronchial epithelial cells are influenced by the state of differentiation. PloS One 5, e8713. 10.1371/journal.pone.0008713 20090947 PMC2806912

[B30] Chen W. Calvo P. A. Malide D. Gibbs J. Schubert U. Bacik I.. (2001). A novel influenza A virus mitochondrial protein that induces cell death. Nat. Med. 7, 1306–1312. 10.1038/nm1201-1306 11726970

[B31] Chen M. C. Lin C. F. Lei H. Y. Lin S. C. Liu H. S. Yeh T. M.. (2009). Deletion of the C-terminal region of dengue virus nonstructural protein 1 (NS1) abolishes anti-NS1-mediated platelet dysfunction and bleeding tendency. J. Immunol. 183, 1797–1803. 10.4049/jimmunol.0800672 19592650

[B32] Chiaretti A. Pulitano S. Barone G. Ferrara P. Romano V. Capozzi D.. (2013). IL-1 beta and IL-6 upregulation in children with H1N1 influenza virus infection. Mediators Inflammation 2013, 495848. 10.1155/2013/495848 PMC3657430 23737648

[B33] Chu Y. T. Wan S. W. Anderson R. Lin Y. S. (2015). Mast cell-macrophage dynamics in modulation of dengue virus infection in skin. Immunology 146, 163–172. 10.1111/imm.12492 26059780 PMC4552511

[B34] Collington S. J. Hallgren J. Pease J. E. Jones T. G. Rollins B. J. Westwick J.. (2010). The role of the CCL2/CCR2 axis in mouse mast cell migration in vitro and in vivo. J. Immunol. 184, 6114–6123. 10.4049/jimmunol.0904177 20427772 PMC2956277

[B35] Da Silva E. Z. Jamur M. C. Oliver C. (2014). Mast cell function: a new vision of an old cell. J. Histochem. Cytochem. 62, 698–738. 10.1369/0022155414545334 25062998 PMC4230976

[B36] Damjanovic D. Divangahi M. Kugathasan K. Small C. L. Zganiacz A. Brown E. G.. (2011). Negative regulation of lung inflammation and immunopathology by TNF-alpha during acute influenza infection. Am. J. Pathol. 179, 2963–2976. 10.1016/j.ajpath.2011.09.003 22001698 PMC3260802

[B37] De Vries V. C. Noelle R. J. (2010). Mast cell mediators in tolerance. Curr. Opin. Immunol. 22, 643–648. 10.1016/j.coi.2010.08.015 20884193 PMC3809014

[B38] Denney L. Branchett W. Gregory L. G. Oliver R. A. Lloyd C. M. (2018). Epithelial-derived TGF-beta1 acts as a pro-viral factor in the lung during influenza A infection. Mucosal Immunol. 11, 523–535. 10.1038/mi.2017.77 29067998 PMC5797694

[B39] Denney L. Ho L. P. (2018). The role of respiratory epithelium in host defence against influenza virus infection. BioMed. J. 41, 218–233. 10.1016/j.bj.2018.08.004 30348265 PMC6197993

[B40] Depinay N. Hacini F. Beghdadi W. Peronet R. Mecheri S. (2006). Mast cell-dependent down-regulation of antigen-specific immune responses by mosquito bites. J. Immunol. 176, 4141–4146. 10.4049/jimmunol.176.7.4141 16547250

[B41] Dienz O. Rud J. G. Eaton S. M. Lanthier P. A. Burg E. Drew A.. (2012). Essential role of IL-6 in protection against H1N1 influenza virus by promoting neutrophil survival in the lung. Mucosal Immunol. 5, 258–266. 10.1038/mi.2012.2 22294047 PMC3328598

[B42] Dinarello C. A. (2019). The IL-1 family of cytokines and receptors in rheumatic diseases. Nat. Rev. Rheumatol. 15, 612–632. 10.1038/s41584-019-0277-8 31515542

[B43] Dobson J. Whitley R. J. Pocock S. Monto A. S. (2015). Oseltamivir treatment for influenza in adults: a meta-analysis of randomised controlled trials. Lancet 385, 1729–1737. 10.1016/S0140-6736(14)62449-1 25640810

[B44] Domenico J. Lucas J. J. Fujita M. Gelfand E. W. (2012). Susceptibility to vaccinia virus infection and spread in mice is determined by age at infection, allergen sensitization and mast cell status. Int. Arch. Allergy Immunol. 158, 196–205. 10.1159/000330647 22286752 PMC3291886

[B45] Dong G. Peng C. Luo J. Wang C. Han L. Wu B.. (2015). Adamantane-resistant influenza a viruses in the world, (1902-2013): frequency and distribution of M2 gene mutations. PloS One 10, e0119115. 10.1371/journal.pone.0119115 25768797 PMC4358984

[B46] Drube S. Kraft F. Dudeck J. Muller A. L. Weber F. Gopfert C.. (2016). MK2/3 Are Pivotal for IL-33-Induced and Mast Cell-Dependent Leukocyte Recruitment and the Resulting Skin Inflammation. J. Immunol. 197, 3662–3668. 10.4049/jimmunol.1600658 27694493

[B47] Dudeck A. Dudeck J. Scholten J. Petzold A. Surianarayanan S. Kohler A.. (2011). Mast cells are key promoters of contact allergy that mediate the adjuvant effects of haptens. Immunity 34, 973–984. 10.1016/j.immuni.2011.03.028 21703544

[B48] Dutta A. Huang C. T. Chen T. C. Lin C. Y. Chiu C. H. Lin Y. C.. (2015). IL-10 inhibits neuraminidase-activated TGF-beta and facilitates Th1 phenotype during early phase of infection. Nat. Commun. 6, 6374. 10.1038/ncomms7374 25728041

[B49] Eisenmesser E. Z. Gottschlich A. Redzic J. S. Paukovich N. Nix J. C. Azam T.. (2019). Interleukin-37 monomer is the active form for reducing innate immunity. Proc. Natl. Acad. Sci. U.S.A. 116, 5514–5522. 10.1073/pnas.1819672116 30819901 PMC6431183

[B50] Fernando J. Faber T. W. Pullen N. A. Falanga Y. T. Kolawole E. M. Oskeritzian C. A.. (2013). Genotype-dependent effects of TGF-beta1 on mast cell function: targeting the Stat5 pathway. J. Immunol. 191, 4505–4513. 10.4049/jimmunol.1202723 24068671 PMC3846427

[B51] Finlay C. M. Cunningham K. T. Doyle B. Mills K. H. G. (2020). IL-33-Stimulated Murine Mast Cells Polarize Alternatively Activated Macrophages, Which Suppress T Cells That Mediate Experimental Autoimmune Encephalomyelitis. J. Immunol. 205, 1909–1919. 10.4049/jimmunol.1901321 32859729

[B52] Fishman D. Faulds G. Jeffery R. Mohamed-Ali V. Yudkin J. S. Humphries S.. (1998). The effect of novel polymorphisms in the interleukin-6 (IL-6) gene on IL-6 transcription and plasma IL-6 levels, and an association with systemic-onset juvenile chronic arthritis. J. Clin. Invest. 102, 1369–1376. 10.1172/JCI2629 9769329 PMC508984

[B53] Fournie G. Guitian J. Desvaux S. Cuong V. C. Dung Do H. Pfeiffer D. U.. (2013). Interventions for avian influenza A (H5N1) risk management in live bird market networks. Proc. Natl. Acad. Sci. U.S.A. 110, 9177–9182. 10.1073/pnas.1220815110 23650388 PMC3670392

[B54] Fujikura D. Miyazaki T. (2018). Programmed Cell Death in the Pathogenesis of Influenza. Int. J. Mol. Sci. 19, 2065–2079. 10.3390/ijms19072065 PMC6073994 30012970

[B55] Galli S. J. Grimbaldeston M. Tsai M. (2008). Immunomodulatory mast cells: negative, as well as positive, regulators of immunity. Nat. Rev. Immunol. 8, 478–486. 10.1038/nri2327 18483499 PMC2855166

[B56] Galli S. J. Gaudenzio N. Tsai M. (2020). Mast Cells in Inflammation and Disease: Recent Progress and Ongoing Concerns. Annu. Rev. Immunol. 38, 49–77. 10.1146/annurev-immunol-071719-094903 32340580

[B57] Ganeshan K. Johnston L. K. Bryce P. J. (2013). TGF-beta1 limits the onset of innate lung inflammation by promoting mast cell-derived IL-6. J. Immunol. 190, 5731–5738. 10.4049/jimmunol.1203362 23630359 PMC3725733

[B58] Ganeshan K. Bryce P. J. (2012). Regulatory T cells enhance mast cell production of IL-6 via surface-bound TGF-beta. J. Immunol. 188, 594–603. 10.4049/jimmunol.1102389 22156492 PMC3253181

[B59] Gaudenzio N. Marichal T. Galli S. J. Reber L. L. (2018). Genetic and Imaging Approaches Reveal Pro-Inflammatory and Immunoregulatory Roles of Mast Cells in Contact Hypersensitivity. Front. Immunol. 9, 1275. 10.3389/fimmu.2018.01275 29922295 PMC5996070

[B60] Godfraind C. Louahed J. Faulkner H. Vink A. Warnier G. Grencis R.. (1998). Intraepithelial infiltration by mast cells with both connective tissue-type and mucosal-type characteristics in gut, trachea, and kidneys of IL-9 transgenic mice. J. Immunol. 160, 3989–3996. 9558107

[B61] Gordon J. R. Galli S. J. (1990). Mast cells as a source of both preformed and immunologically inducible TNF-alpha/cachectin. Nature 346, 274–276. 10.1038/346274a0 2374592

[B62] Gordon J. R. Galli S. J. (1994). Promotion of mouse fibroblast collagen gene expression by mast cells stimulated via the Fc epsilon RI. Role for mast cell-derived transforming growth factor beta and tumor necrosis factor alpha. J. Exp. Med. 180, 2027–2037. 10.1084/jem.180.6.2027 7964480 PMC2191776

[B63] Graham A. C. Hilmer K. M. Zickovich J. M. Obar J. J. (2013). Inflammatory response of mast cells during influenza A virus infection is mediated by active infection and RIG-I signaling. J. Immunol. 190, 4676–4684. 10.4049/jimmunol.1202096 23526820 PMC3633673

[B64] Graham A. C. Temple R. M. Obar J. J. (2015). Mast cells and influenza a virus: association with allergic responses and beyond. Front. Immunol. 6, 238. 10.3389/fimmu.2015.00238 26042121 PMC4435071

[B65] Gri G. Frossi B. D’inca F. Danelli L. Betto E. Mion F.. (2012). Mast cell: an emerging partner in immune interaction. Front. Immunol. 3, 120. 10.3389/fimmu.2012.00120 22654879 PMC3360165

[B66] Grimbaldeston M. A. Nakae S. Kalesnikoff J. Tsai M. Galli S. J. (2007). Mast cell-derived interleukin 10 limits skin pathology in contact dermatitis and chronic irradiation with ultraviolet B. Nat. Immunol. 8, 1095–1104. 10.1038/ni1503 17767162

[B67] Grunewald S. M. Hahn C. Wohlleben G. Teufel M. Major T. Moll H.. (2002). Infection with influenza a virus leads to flu antigen-induced cutaneous anaphylaxis in mice. J. Invest. Dermatol. 118, 645–651. 10.1046/j.1523-1747.2002.01732.x 11918711

[B68] Gubareva L. V. Trujillo A. A. Okomo-Adhiambo M. Mishin V. P. Deyde V. M. Sleeman K.. (2010). Comprehensive assessment of 2009 pandemic influenza A (H1N1) virus drug susceptibility in vitro. Antivir. Ther. 15, 1151–1159. 10.3851/IMP1678 21149922

[B69] Gutierrez L. Jang M. Zhang T. Akhtari M. Alachkar H. (2018). Midostaurin reduces Regulatory T cells markers in Acute Myeloid Leukemia. Sci. Rep. 8, 17544. 10.1038/s41598-018-35978-0 30510164 PMC6277419

[B70] Hagaman D. D. Okayama Y. D’ambrosio C. Prussin C. Gilfillan A. M. Metcalfe D. D. (2001). Secretion of interleukin-1 receptor antagonist from human mast cells after immunoglobulin E-mediated activation and after segmental antigen challenge. Am. J. Respir. Cell Mol. Biol. 25, 685–691. 10.1165/ajrcmb.25.6.4541 11726393

[B71] Hallgren J. Jones T. G. Abonia J. P. Xing W. Humbles A. Austen K. F.. (2007). Pulmonary CXCR2 regulates VCAM-1 and antigen-induced recruitment of mast cell progenitors. Proc. Natl. Acad. Sci. U.S.A. 104, 20478–20483. 10.1073/pnas.0709651104 18077323 PMC2154456

[B72] Halstead E. S. Umstead T. M. Davies M. L. Kawasawa Y. I. Silveyra P. Howyrlak J.. (2018). GM-CSF overexpression after influenza a virus infection prevents mortality and moderates M1-like airway monocyte/macrophage polarization. Respir. Res. 19, 3. 10.1186/s12931-017-0708-5 29304863 PMC5756339

[B73] Hamada H. Bassity E. Flies A. Strutt T. M. Garcia-Hernandez Mde L. Mckinstry K. K.. (2013). Multiple redundant effector mechanisms of CD8+ T cells protect against influenza infection. J. Immunol. 190, 296–306. 10.4049/jimmunol.1200571 23197262 PMC3864858

[B74] Hamilton J. A. (2019). GM-CSF-Dependent Inflammatory Pathways. Front. Immunol. 10, 2055. 10.3389/fimmu.2019.02055 31552022 PMC6737278

[B75] Han J. Zhang N. Zhang P. Yang C. Jin M. Yang J.. (2014). Th2-type inflammation under conditions of pre-existing chronic disease is associated with liver damage in patients with avian influenza H7N9 virus. Microbes Infect. 16, 672–677. 10.1016/j.micinf.2014.04.002 24769417

[B76] Han D. Wei T. Zhang S. Wang M. Tian H. Cheng J.. (2016). The therapeutic effects of sodium cromoglycate against influenza A virus H5N1 in mice. Influenza Other Respir. Viruses 10, 57–66. 10.1111/irv.12334 26176755 PMC4687497

[B77] Hart P. H. Grimbaldeston M. A. Swift G. J. Jaksic A. Noonan F. P. Finlay-Jones J. J. (1998). Dermal mast cells determine susceptibility to ultraviolet B-induced systemic suppression of contact hypersensitivity responses in mice. J. Exp. Med. 187, 2045–2053. 10.1084/jem.187.12.2045 9625764 PMC2212357

[B78] Heinrich P. C. Behrmann I. Haan S. Hermanns H. M. Muller-Newen G. Schaper F. (2003). Principles of interleukin (IL)-6-type cytokine signalling and its regulation. Biochem. J. 374, 1–20. 10.1042/bj20030407 12773095 PMC1223585

[B79] Hendrix S. Kramer P. Pehl D. Warnke K. Boato F. Nelissen S.. (2013). Mast cells protect from post-traumatic brain inflammation by the mast cell-specific chymase mouse mast cell protease-4. FASEB J. 27, 920–929. 10.1096/fj.12-204800 23193170

[B80] Herold S. Von Wulffen W. Steinmueller M. Pleschka S. Kuziel W. A. Mack M.. (2006). Alveolar epithelial cells direct monocyte transepithelial migration upon influenza virus infection: impact of chemokines and adhesion molecules. J. Immunol. 177, 1817–1824. 10.4049/jimmunol.177.3.1817 16849492

[B81] Hu Y. Jin Y. Han D. Zhang G. Cao S. Xie J.. (2012). Mast cell-induced lung injury in mice infected with H5N1 influenza virus. J. Virol. 86, 3347–3356. 10.1128/JVI.06053-11 22238293 PMC3302317

[B82] Huang Y. C. Shieh H. R. Chen Y. J. (2010). Midostaurin (PKC412) modulates differentiation and maturation of human myeloid dendritic cells. Toxicol. In Vitro 24, 1705–1710. 10.1016/j.tiv.2010.05.015 20685248

[B83] Huang F. F. Barnes P. F. Feng Y. Donis R. Chroneos Z. C. Idell S.. (2011). GM-CSF in the lung protects against lethal influenza infection. Am. J. Respir. Crit. Care Med. 184, 259–268. 10.1164/rccm.201012-2036OC 21474645 PMC6938174

[B84] Hultner L. Druez C. Moeller J. Uyttenhove C. Schmitt E. Rude E.. (1990). Mast cell growth-enhancing activity (MEA) is structurally related and functionally identical to the novel mouse T cell growth factor P40/TCGFIII (interleukin 9). Eur. J. Immunol. 20, 1413–1416. 10.1002/eji.1830200632 2115002

[B85] Hultner L. Kolsch S. Stassen M. Kaspers U. Kremer J. P. Mailhammer R.. (2000). In activated mast cells, IL-1 up-regulates the production of several Th2-related cytokines including IL-9. J. Immunol. 164, 5556–5563. 10.4049/jimmunol.164.11.5556 10820229

[B86] Hunter C. A. Jones S. A. (2015). IL-6 as a keystone cytokine in health and disease. Nat. Immunol. 16, 448–457. 10.1038/ni.3153 25898198

[B87] Hurst S. M. Wilkinson T. S. Mcloughlin R. M. Jones S. Horiuchi S. Yamamoto N.. (2001). Il-6 and its soluble receptor orchestrate a temporal switch in the pattern of leukocyte recruitment seen during acute inflammation. Immunity 14, 705–714. 10.1016/S1074-7613(01)00151-0 11420041

[B88] Hussell T. Pennycook A. Openshaw P. J. (2001). Inhibition of tumor necrosis factor reduces the severity of virus-specific lung immunopathology. Eur. J. Immunol. 31, 2566–2573. 10.1002/1521-4141(200109)31:9<2566::AID-IMMU2566>3.0.CO;2-L 11536154

[B89] Ishige T. Igarashi Y. Hatori R. Tatsuki M. Sasahara Y. Takizawa T.. (2018). IL-10RA Mutation as a Risk Factor of Severe Influenza-Associated Encephalopathy: A Case Report. Pediatrics 141, 2017–3548. 10.1542/peds.2017-3548 29724880

[B90] Iuliano A. D. Roguski K. M. Chang H. H. Muscatello D. J. Palekar R. Tempia S.. (2018). Estimates of global seasonal influenza-associated respiratory mortality: a modelling study. Lancet 391, 1285–1300. 10.1016/S0140-6736(17)33293-2 29248255 PMC5935243

[B91] Jang H. Boltz D. Mcclaren J. Pani A. K. Smeyne M. Korff A.. (2012). Inflammatory effects of highly pathogenic H5N1 influenza virus infection in the CNS of mice. J. Neurosci. 32, 1545–1559. 10.1523/JNEUROSCI.5123-11.2012 22302798 PMC3307392

[B92] Jones S. A. Horiuchi S. Topley N. Yamamoto N. Fuller G. M. (2001). The soluble interleukin 6 receptor: mechanisms of production and implications in disease. FASEB J. 15, 43–58. 10.1096/fj.99-1003rev 11149892

[B93] Jones T. G. Hallgren J. Humbles A. Burwell T. Finkelman F. D. Alcaide P.. (2009). Antigen-induced increases in pulmonary mast cell progenitor numbers depend on IL-9 and CD1d-restricted NKT cells. J. Immunol. 183, 5251–5260. 10.4049/jimmunol.0901471 19783672 PMC2782612

[B94] Jones S. A. (2005). Directing transition from innate to acquired immunity: defining a role for IL-6. J. Immunol. 175, 3463–3468. 10.4049/jimmunol.175.6.3463 16148087

[B95] Jones S. A. Jenkins B. J. (2018). Recent insights into targeting the IL-6 cytokine family in inflammatory diseases and cancer. Nat. Rev. Immunol. 18, 773–789. 10.1038/s41577-018-0066-7 30254251

[B96] Josset L. Belser J. A. Pantin-Jackwood M. J. Chang J. H. Chang S. T. Belisle S. E.. (2012). Implication of inflammatory macrophages, nuclear receptors, and interferon regulatory factors in increased virulence of pandemic 2009 H1N1 influenza A virus after host adaptation. J. Virol. 86, 7192–7206. 10.1128/JVI.00563-12 22532695 PMC3416346

[B97] Kandere-Grzybowska K. Letourneau R. Kempuraj D. Donelan J. Poplawski S. Boucher W.. (2003). IL-1 induces vesicular secretion of IL-6 without degranulation from human mast cells. J. Immunol. 171, 4830–4836. 10.4049/jimmunol.171.9.4830 14568962

[B98] Kandere-Grzybowska K. Kempuraj D. Cao J. Cetrulo C. L. Theoharides T. C. (2006). Regulation of IL-1-induced selective IL-6 release from human mast cells and inhibition by quercetin. Br. J. Pharmacol. 148, 208–215. 10.1038/sj.bjp.0706695 16532021 PMC1617055

[B99] Kaplan M. H. Hufford M. M. Olson M. R. (2015). The development and in vivo function of T helper 9 cells. Nat. Rev. Immunol. 15, 295–307. 10.1038/nri3824 25848755 PMC4445728

[B100] Karaman M. W. Herrgard S. Treiber D. K. Gallant P. Atteridge C. E. Campbell B. T.. (2008). A quantitative analysis of kinase inhibitor selectivity. Nat. Biotechnol. 26, 127–132. 10.1038/nbt1358 18183025

[B101] Kasamon Y. L. Ko C. W. Subramaniam S. Ma L. Yang Y. Nie L.. (2018). FDA Approval Summary: Midostaurin for the Treatment of Advanced Systemic Mastocytosis. Oncologist 23, 1511–1519. 10.1634/theoncologist.2018-0222 30115735 PMC6292539

[B102] Kearley J. Erjefalt J. S. Andersson C. Benjamin E. Jones C. P. Robichaud A.. (2011). IL-9 governs allergen-induced mast cell numbers in the lung and chronic remodeling of the airways. Am. J. Respir. Crit. Care Med. 183, 865–875. 10.1164/rccm.200909-1462OC 20971830 PMC3385369

[B103] Kim H. M. Lee Y. W. Lee K. J. Kim H. S. Cho S. W. Van Rooijen N.. (2008). Alveolar macrophages are indispensable for controlling influenza viruses in lungs of pigs. J. Virol. 82, 4265–4274. 10.1128/JVI.02602-07 18287245 PMC2293066

[B104] Kitamura Y. Shimada M. Hatanaka K. Miyano Y. (1977). Development of mast cells from grafted bone marrow cells in irradiated mice. Nature 268, 442–443. 10.1038/268442a0 331117

[B105] Kotenko S. V. Krause C. D. Izotova L. S. Pollack B. P. Wu W. Pestka S. (1997). Identification and functional characterization of a second chain of the interleukin-10 receptor complex. EMBO J. 16, 5894–5903. 10.1093/emboj/16.19.5894 9312047 PMC1170220

[B106] Krammer F. Smith G. J. D. Fouchier R. Peiris M. Kedzierska K. Doherty P. C.. (2018). Influenza. Nat. Rev. Dis. Primers 4, 3. 10.1038/s41572-018-0002-y 29955068 PMC7097467

[B107] Krystel-Whittemore M. Dileepan K. N. Wood J. G. (2015). Mast Cell: A Multi-Functional Master Cell. Front. Immunol. 6, 620. 10.3389/fimmu.2015.00620 26779180 PMC4701915

[B108] Kulka M. Alexopoulou L. Flavell R. A. Metcalfe D. D. (2004). Activation of mast cells by double-stranded RNA: evidence for activation through Toll-like receptor 3. J. Allergy Clin. Immunol. 114, 174–182. 10.1016/j.jaci.2004.03.049 15241362

[B109] Kuschert G. S. Coulin F. Power C. A. Proudfoot A. E. Hubbard R. E. Hoogewerf A. J.. (1999). Glycosaminoglycans interact selectively with chemokines and modulate receptor binding and cellular responses. Biochemistry 38, 12959–12968. 10.1021/bi990711d 10504268

[B110] Kwong P. D. Dekosky B. J. Ulmer J. B. (2020). Antibody-guided structure-based vaccines. Semin. Immunol., 50, 101428. 10.1016/j.smim.2020.101428 33246736

[B111] Lam W. Y. Tang J. W. Yeung A. C. Chiu L. C. Sung J. J. Chan P. K. (2008). Avian influenza virus A/HK/483/97(H5N1) NS1 protein induces apoptosis in human airway epithelial cells. J. Virol. 82, 2741–2751. 10.1128/JVI.01712-07 18199656 PMC2258969

[B112] Lam W. Y. Yeung A. C. Chu I. M. Chan P. K. (2010). Profiles of cytokine and chemokine gene expression in human pulmonary epithelial cells induced by human and avian influenza viruses. Virol. J. 7, 344. 10.1186/1743-422X-7-344 21108843 PMC3002310

[B113] Lang V. R. Englbrecht M. Rech J. Nusslein H. Manger K. Schuch F.. (2012). Risk of infections in rheumatoid arthritis patients treated with tocilizumab. Rheumatol. (Oxford) 51, 852–857. 10.1093/rheumatology/ker223 21865281

[B114] Lefrancais E. Duval A. Mirey E. Roga S. Espinosa E. Cayrol C.. (2014). Central domain of IL-33 is cleaved by mast cell proteases for potent activation of group-2 innate lymphoid cells. Proc. Natl. Acad. Sci. U.S.A. 111, 15502–15507. 10.1073/pnas.1410700111 25313073 PMC4217470

[B115] Li Y. Shi Y. Mccaw L. Li Y. J. Zhu F. Gorczynski R.. (2015). Microenvironmental interleukin-6 suppresses toll-like receptor signaling in human leukemia cells through miR-17/19A. Blood 126, 766–778. 10.1182/blood-2014-12-618678 26041742

[B116] Li S. Amo-Aparicio J. Neff C. P. Tengesdal I. W. Azam T. Palmer B. E.. (2019). Role for nuclear interleukin-37 in the suppression of innate immunity. Proc. Natl. Acad. Sci. U.S.A. 116, 4456–4461. 10.1073/pnas.1821111116 30792349 PMC6410848

[B117] Lindstedt K. A. Wang Y. Shiota N. Saarinen J. Hyytiainen M. Kokkonen J. O.. (2001). Activation of paracrine TGF-beta1 signaling upon stimulation and degranulation of rat serosal mast cells: a novel function for chymase. FASEB J. 15, 1377–1388. 10.1096/fj.00-0273com 11387235

[B118] Lindstrom S. E. Cox N. J. Klimov A. (2004). Genetic analysis of human H2N2 and early H3N2 influenza viruse -1972: evidence for genetic divergence and multiple reassortment events. Virology 328, 101–119. 10.1016/j.virol.2004.06.009 15380362

[B119] Liu H. Golebiewski L. Dow E. C. Krug R. M. Javier R. T. Rice A. P. (2010). The ESEV PDZ-binding motif of the avian influenza A virus NS1 protein protects infected cells from apoptosis by directly targeting Scribble. J. Virol. 84, 11164–11174. 10.1128/JVI.01278-10 20702615 PMC2953166

[B120] Liu B. Meng D. Wei T. Zhang S. Hu Y. Wang M. (2014). Apoptosis and pro-inflammatory cytokine response of mast cells induced by influenza A viruses. PloS One 9, e100109. 10.1371/journal.pone.0100109 24923273 PMC4055757

[B121] Liu Q. Zhou Y. H. Yang Z. Q. (2016). The cytokine storm of severe influenza and development of immunomodulatory therapy. Cell Mol. Immunol. 13, 3–10. 10.1038/cmi.2015.74 26189369 PMC4711683

[B122] Marcet C. W. St Laurent C. D. Moon T. C. Singh N. Befus A. D. (2013). Limited replication of influenza A virus in human mast cells. Immunol. Res. 56, 32–43. 10.1007/s12026-012-8377-4 23055084

[B123] Marichal T. Starkl P. Reber L. L. Kalesnikoff J. Oettgen H. C. Tsai M.. (2013). A beneficial role for immunoglobulin E in host defense against honeybee venom. Immunity 39, 963–975. 10.1016/j.immuni.2013.10.005 24210352 PMC4164235

[B124] Marshall J. S. Portales-Cervantes L. Leong E. (2019). Mast Cell Responses to Viruses and Pathogen Products. Int. J. Mol. Sci. 20, 4241–4259. 10.3390/ijms20174241 PMC6747121 31480219

[B125] Martin-Loeches I. Sole-Violan J. Rodriguez De Castro F. Garcia-Laorden M. I. Borderias L. Blanquer J.. (2012). Variants at the promoter of the interleukin-6 gene are associated with severity and outcome of pneumococcal community-acquired pneumonia. Intensive Care Med. 38, 256–262. 10.1007/s00134-011-2406-y 22113815

[B126] Matsukura S. Kokubu F. Kubo H. Tomita T. Tokunaga H. Kadokura M.. (1998). Expression of RANTES by normal airway epithelial cells after influenza virus A infection. Am. J. Respir. Cell Mol. Biol. 18, 255–264. 10.1165/ajrcmb.18.2.2822 9476913

[B127] Matsuzawa S. Sakashita K. Kinoshita T. Ito S. Yamashita T. Koike K. (2003). IL-9 enhances the growth of human mast cell progenitors under stimulation with stem cell factor. J. Immunol. 170, 3461–3467. 10.4049/jimmunol.170.7.3461 12646606

[B128] Mckinstry K. K. Strutt T. M. Buck A. Curtis J. D. Dibble J. P. Huston G.. (2009). IL-10 deficiency unleashes an influenza-specific Th17 response and enhances survival against high-dose challenge. J. Immunol. 182, 7353–7363. 10.4049/jimmunol.0900657 19494257 PMC2724021

[B129] Metz M. Piliponsky A. M. Chen C. C. Lammel V. Abrink M. Pejler G.. (2006). Mast cells can enhance resistance to snake and honeybee venoms. Science 313, 526–530. 10.1126/science.1128877 16873664

[B130] Miller S. J. Hoggat A. M. Faulk W. P. (1998). Heparin regulates ICAM-1 expression in human endothelial cells: an example of non-cytokine-mediated endothelial activation. Thromb. Haemost. 80, 481–487. 10.1055/s-0037-1615233 9759631

[B131] Montier Y. Lorentz A. Kramer S. Sellge G. Schock M. Bauer M.. (2012). Central role of IL-6 and MMP-1 for cross talk between human intestinal mast cells and human intestinal fibroblasts. Immunobiology 217, 912–919. 10.1016/j.imbio.2012.01.003 22356938

[B132] Morrison J. Josset L. Tchitchek N. Chang J. Belser J. A. Swayne D. E.. (2014). H7N9 and other pathogenic avian influenza viruses elicit a three-pronged transcriptomic signature that is reminiscent of 1918 influenza virus and is associated with lethal outcome in mice. J. Virol. 88, 10556–10568. 10.1128/JVI.00570-14 24991006 PMC4178843

[B133] Mukai K. Tsai M. Saito H. Galli S. J. (2018). Mast cells as sources of cytokines, chemokines, and growth factors. Immunol. Rev. 282, 121–150. 10.1111/imr.12634 29431212 PMC5813811

[B134] Munoz S. Rivas-Santiago B. Enciso J. A. (2009). Mycobacterium tuberculosis entry into mast cells through cholesterol-rich membrane microdomains. Scand. J. Immunol. 70, 256–263. 10.1111/j.1365-3083.2009.02295.x 19703015

[B135] Murray D. B. Gardner J. D. Brower G. L. Janicki J. S. (2004). Endothelin-1 mediates cardiac mast cell degranulation, matrix metalloproteinase activation, and myocardial remodeling in rats. Am. J. Physiol. Heart Circ. Physiol. 287, H2295–H2299. 10.1152/ajpheart.00048.2004 15231495

[B136] Muthuri S. G. Venkatesan S. Myles P. R. Leonardi-Bee J. Al Khuwaitir T. S. Al Mamun A.. (2014). Effectiveness of neuraminidase inhibitors in reducing mortality in patients admitted to hospital with influenza A H1N1pdm09 virus infection: a meta-analysis of individual participant data. Lancet Respir. Med. 2, 395–404. 10.1016/S2213-2600(14)70041-4 24815805 PMC6637757

[B137] Nagarkar D. R. Poposki J. A. Comeau M. R. Biyasheva A. Avila P. C. Schleimer R. P.. (2012). Airway epithelial cells activate TH2 cytokine production in mast cells through IL-1 and thymic stromal lymphopoietin. J. Allergy Clin. Immunol. 130, 225–232.e224. 10.1016/j.jaci.2012.04.019 22633328 PMC3387295

[B138] Nelson R. M. Cecconi O. Roberts W. G. Aruffo A. Linhardt R. J. Bevilacqua M. P. (1993). Heparin oligosaccharides bind L- and P-selectin and inhibit acute inflammation. Blood 82, 3253–3258. 10.1182/blood.V82.11.3253.3253 7694675

[B139] Ng K. Raheem J. St Laurent C. D. Marcet C. T. Vliagoftis H. Befus A. D.. (2019). Responses of human mast cells and epithelial cells following exposure to influenza A virus. Antiviral Res. 171, 104566. 10.1016/j.antiviral.2019.104566 31348951

[B140] Norman M. U. Hwang J. Hulliger S. Bonder C. S. Yamanouchi J. Santamaria P.. (2008). Mast cells regulate the magnitude and the cytokine microenvironment of the contact hypersensitivity response. Am. J. Pathol. 172, 1638–1649. 10.2353/ajpath.2008.070559 18467702 PMC2408423

[B141] Obenauer J. C. Denson J. Mehta P. K. Su X. Mukatira S. Finkelstein D. B.. (2006). Large-scale sequence analysis of avian influenza isolates. Science 311, 1576–1580. 10.1126/science.1121586 16439620

[B142] Okoli G. N. Otete H. E. Beck C. R. Nguyen-Van-Tam J. S. (2014). Use of neuraminidase inhibitors for rapid containment of influenza: a systematic review and meta-analysis of individual and household transmission studies. PloS One 9, e113633. 10.1371/journal.pone.0113633 25490762 PMC4260958

[B143] Oldford S. A. Salsman S. P. Portales-Cervantes L. Alyazidi R. Anderson R. Haidl I. D.. (2018). Interferon alpha2 and interferon gamma induce the degranulation independent production of VEGF-A and IL-1 receptor antagonist and other mediators from human mast cells. Immun. Inflammation Dis. 6, 176–189. 10.1002/iid3.211 PMC5818443 29235261

[B144] Ouyang W. Rutz S. Crellin N. K. Valdez P. A. Hymowitz S. G. (2011). Regulation and functions of the IL-10 family of cytokines in inflammation and disease. Annu. Rev. Immunol. 29, 71–109. 10.1146/annurev-immunol-031210-101312 21166540

[B145] Pejler G. Ronnberg E. Waern I. Wernersson S. (2010). Mast cell proteases: multifaceted regulators of inflammatory disease. Blood 115, 4981–4990. 10.1182/blood-2010-01-257287 20233968

[B146] Peters M. Meyer Zum Buschenfelde K. H. Rose-John S. (1996). The function of the soluble IL-6 receptor in vivo. Immunol. Lett. 54, 177–184. 10.1016/S0165-2478(96)02669-7 9052874

[B147] Petersen A. M. Pedersen B. K. (2006). The role of IL-6 in mediating the anti-inflammatory effects of exercise. J. Physiol. Pharmacol. 57 (Suppl 10), 43–51. 10.1249/00005768-200605001-00226 17242490

[B148] Piliponsky A. M. Chen C. C. Rios E. J. Treuting P. M. Lahiri A. Abrink M.. (2012). The chymase mouse mast cell protease 4 degrades TNF, limits inflammation, and promotes survival in a model of sepsis. Am. J. Pathol. 181, 875–886. 10.1016/j.ajpath.2012.05.013 22901752 PMC3432424

[B149] Plum T. Wang X. Rettel M. Krijgsveld J. Feyerabend T. B. Rodewald H. R. (2020). Human Mast Cell Proteome Reveals Unique Lineage, Putative Functions, and Structural Basis for Cell Ablation. Immunity 52, 404–416.e405. 10.1016/j.immuni.2020.01.012 32049054

[B150] Qi F. Liu M. Li F. Lv Q. Wang G. Gong S.. (2019). Interleukin-37 Ameliorates Influenza Pneumonia by Attenuating Macrophage Cytokine Production in a MAPK-Dependent Manner. Front. Microbiol. 10, 2482. 10.3389/fmicb.2019.02482 31736917 PMC6831648

[B151] Ramos I. Fernandez-Sesma A. (2015). Modulating the Innate Immune Response to Influenza A Virus: Potential Therapeutic Use of Anti-Inflammatory Drugs. Front. Immunol. 6, 361. 10.3389/fimmu.2015.00361 26257731 PMC4507467

[B152] Rathore A. P. St John A. L. (2020). Protective and pathogenic roles for mast cells during viral infections. Curr. Opin. Immunol. 66, 74–81. 10.1016/j.coi.2020.05.003 32563779 PMC7301783

[B153] Reber L. L. Sibilano R. Mukai K. Galli S. J. (2015). Potential effector and immunoregulatory functions of mast cells in mucosal immunity. Mucosal Immunol. 8, 444–463. 10.1038/mi.2014.131 25669149 PMC4739802

[B154] Reber L. L. Sibilano R. Starkl P. Roers A. Grimbaldeston M. A. Tsai M.. (2017) Imaging protective mast cells in living mice during severe contact hypersensitivity. JCI Insight 2, 92900–92912. 10.1172/jci.insight.92900 28469089 PMC5414565

[B155] Reeh H. Rudolph N. Billing U. Christen H. Streif S. Bullinger E.. (2019). Response to IL-6 trans- and IL-6 classic signalling is determined by the ratio of the IL-6 receptor alpha to gp130 expression: fusing experimental insights and dynamic modelling. Cell Commun. Signal 17, 46. 10.1186/s12964-019-0356-0 31101051 PMC6525395

[B156] Riley J. F. (1953). Histamine in tissue mast cells. Science 118, 332. 10.1126/science.118.3064.332 13089701

[B157] Rodewald H. R. Feyerabend T. B. (2012). Widespread immunological functions of mast cells: fact or fiction? Immunity 37, 13–24. 10.1016/j.immuni.2012.07.007 22840840

[B158] Rosler B. Herold S. (2016). Lung epithelial GM-CSF improves host defense function and epithelial repair in influenza virus pneumonia-a new therapeutic strategy? Mol. Cell Pediatr. 3, 29. 10.1186/s40348-016-0055-5 27480877 PMC4969252

[B159] Roy A. Ganesh G. Sippola H. Bolin S. Sawesi O. Dagalv A.. (2014). Mast cell chymase degrades the alarmins heat shock protein 70, biglycan, HMGB1, and interleukin-33 (IL-33) and limits danger-induced inflammation. J. Biol. Chem. 289, 237–250. 10.1074/jbc.M112.435156 24257755 PMC3879547

[B160] Royer D. J. Zheng M. Conrady C. D. Carr D. J. (2015). Granulocytes in Ocular HSV-1 Infection: Opposing Roles of Mast Cells and Neutrophils. Invest. Ophthalmol. Vis. Sci. 56, 3763–3775. 10.1167/iovs.15-16900 26066745 PMC4468912

[B161] Salomon R. Hoffmann E. Webster R. G. (2007). Inhibition of the cytokine response does not protect against lethal H5N1 influenza infection. Proc. Natl. Acad. Sci. U.S.A. 104, 12479–12481. 10.1073/pnas.0705289104 17640882 PMC1924467

[B162] Saluja R. Delin I. Nilsson G. P. Adner M. (2012). FcepsilonR1-mediated mast cell reactivity is amplified through prolonged Toll-like receptor-ligand treatment. PloS One 7, e43547. 10.1371/journal.pone.0043547 22916277 PMC3420882

[B163] Sanders C. J. Doherty P. C. Thomas P. G. (2011). Respiratory epithelial cells in innate immunity to influenza virus infection. Cell Tissue Res. 343, 13–21. 10.1007/s00441-010-1043-z 20848130

[B164] Sandig H. Bulfone-Paus S. (2012). TLR signaling in mast cells: common and unique features. Front. Immunol. 3, 185. 10.3389/fimmu.2012.00185 22783258 PMC3389341

[B165] Schindler R. Mancilla J. Endres S. Ghorbani R. Clark S. C. Dinarello C. A. (1990). Correlations and interactions in the production of interleukin-6 (IL-6), IL-1, and tumor necrosis factor (TNF) in human blood mononuclear cells: IL-6 suppresses IL-1 and TNF. Blood 75, 40–47. 10.1182/blood.V75.1.40.40 2294996

[B166] Schneider L. A. Schlenner S. M. Feyerabend T. B. Wunderlin M. Rodewald H. R. (2007). Molecular mechanism of mast cell mediated innate defense against endothelin and snake venom sarafotoxin. J. Exp. Med. 204, 2629–2639. 10.1084/jem.20071262 17923505 PMC2118486

[B167] Sehra S. Yao W. Nguyen E. T. Glosson-Byers N. L. Akhtar N. Zhou B.. (2015). TH9 cells are required for tissue mast cell accumulation during allergic inflammation. J. Allergy Clin. Immunol. 136, 433–440.e431. 10.1016/j.jaci.2015.01.021 25746972 PMC4530056

[B168] Shale M. Czub M. Kaplan G. G. Panaccione R. Ghosh S. (2010). Anti-tumor necrosis factor therapy and influenza: keeping it in perspective. Therap. Adv. Gastroenterol. 3, 173–177. 10.1177/1756283X10366368 PMC3002575 21180599

[B169] Shi X. Zhou W. Huang H. Zhu H. Zhou P. Zhu H.. (2013). Inhibition of the inflammatory cytokine tumor necrosis factor-alpha with etanercept provides protection against lethal H1N1 influenza infection in mice. Crit. Care 17, R301. 10.1186/cc13171 24373231 PMC4057515

[B170] Short K. R. Kroeze E. Fouchier R. Kuiken T. (2014). Pathogenesis of influenza-induced acute respiratory distress syndrome. Lancet Infect. Dis. 14, 57–69. 10.1016/S1473-3099(13)70286-X 24239327

[B171] Sibilano R. Frossi B. Pucillo C. E. (2014). Mast cell activation: a complex interplay of positive and negative signaling pathways. Eur. J. Immunol. 44, 2558–2566. 10.1002/eji.201444546 25066089

[B172] Silver J. S. Hunter C. A. (2010). gp130 at the nexus of inflammation, autoimmunity, and cancer. J. Leukoc. Biol. 88, 1145–1156. 10.1189/jlb.0410217 20610800 PMC2996896

[B173] Smith G. J. Vijaykrishna D. Bahl J. Lycett S. J. Worobey M. Pybus O. G.. (2009). Origins and evolutionary genomics of the 2009 swine-origin H1N1 influenza A epidemic. Nature 459, 1122–1125. 10.1038/nature08182 19516283

[B174] Sprenger H. Meyer R. G. Kaufmann A. Bussfeld D. Rischkowsky E. Gemsa D. (1996). Selective induction of monocyte and not neutrophil-attracting chemokines after influenza A virus infection. J. Exp. Med. 184, 1191–1196. 10.1084/jem.184.3.1191 9064338 PMC2192790

[B175] St John A. L. Rathore A. P. Yap H. Ng M. L. Metcalfe D. D. Vasudevan S. G.. (2011). Immune surveillance by mast cells during dengue infection promotes natural killer (NK) and NKT-cell recruitment and viral clearance. Proc. Natl. Acad. Sci. U.S.A. 108, 9190–9195. 10.1073/pnas.1105079108 21576486 PMC3107258

[B176] St John A. L. Rathore A. P. Raghavan B. Ng M. L. Abraham S. N. (2013). Contributions of mast cells and vasoactive products, leukotrienes and chymase, to dengue virus-induced vascular leakage. Elife 2, e00481. 10.7554/eLife.00481.015 23638300 PMC3639510

[B177] Starkl P. Marichal T. Gaudenzio N. Reber L. L. Sibilano R. Tsai M.. (2016). IgE antibodies, FcepsilonRIalpha, and IgE-mediated local anaphylaxis can limit snake venom toxicity. J. Allergy Clin. Immunol. 137, 246–257 e211. 10.1016/j.jaci.2015.08.005 26410782 PMC4715494

[B178] Stassen M. Arnold M. Hultner L. Muller C. Neudorfl C. Reineke T.. (2000). Murine bone marrow-derived mast cells as potent producers of IL-9: costimulatory function of IL-10 and kit ligand in the presence of IL-1. J. Immunol. 164, 5549–5555. 10.4049/jimmunol.164.11.5549 10820228

[B179] Subramaniam R. Hillberry Z. Chen H. Feng Y. Fletcher K. Neuenschwander P.. (2015). Delivery of GM-CSF to Protect against Influenza Pneumonia. PloS One 10, e0124593. 10.1371/journal.pone.0124593 25923215 PMC4414562

[B180] Sun J. Madan R. Karp C. L. Braciale T. J. (2009). Effector T cells control lung inflammation during acute influenza virus infection by producing IL-10. Nat. Med. 15, 277–284. 10.1038/nm.1929 19234462 PMC2693210

[B181] Sun K. Torres L. Metzger D. W. (2010). A detrimental effect of interleukin-10 on protective pulmonary humoral immunity during primary influenza A virus infection. J. Virol. 84, 5007–5014. 10.1128/JVI.02408-09 20200252 PMC2863832

[B182] Syenina A. Jagaraj C. J. Aman S. A. Sridharan A. St John A. L. (2015). Dengue vascular leakage is augmented by mast cell degranulation mediated by immunoglobulin Fcgamma receptors. Elife 4. 10.7554/eLife.05291.008 PMC4362203 25783751

[B183] Takeda K. Clausen B. E. Kaisho T. Tsujimura T. Terada N. Forster I.. (1999). Enhanced Th1 activity and development of chronic enterocolitis in mice devoid of Stat3 in macrophages and neutrophils. Immunity 10, 39–49. 10.1016/S1074-7613(00)80005-9 10023769

[B184] Tanaka T. Narazaki M. Kishimoto T. (2014). IL-6 in inflammation, immunity, and disease. Cold Spring Harb. Perspect. Biol. 6, 016295–016312. 10.1101/cshperspect.a016295 PMC4176007 25190079

[B185] Tanaka T. Narazaki M. Kishimoto T. (2018). Interleukin (IL-6) Immunotherapy. Cold Spring Harb. Perspect. Biol. 10, 028456–028472. 10.1101/cshperspect.a028456 PMC6071487 28778870

[B186] Taracanova A. Alevizos M. Karagkouni A. Weng Z. Norwitz E. Conti P.. (2017). SP and IL-33 together markedly enhance TNF synthesis and secretion from human mast cells mediated by the interaction of their receptors. Proc. Natl. Acad. Sci. U.S.A. 114, E4002–E4009. 10.1073/pnas.1524845114 28461492 PMC5441798

[B187] Teijaro J. R. Walsh K. B. Cahalan S. Fremgen D. M. Roberts E. Scott F.. (2011). Endothelial cells are central orchestrators of cytokine amplification during influenza virus infection. Cell 146, 980–991. 10.1016/j.cell.2011.08.015 21925319 PMC3176439

[B188] Terry C. F. Loukaci V. Green F. R. (2000). Cooperative influence of genetic polymorphisms on interleukin 6 transcriptional regulation. J. Biol. Chem. 275, 18138–18144. 10.1074/jbc.M000379200 10747905

[B189] Theoharides T. C. Tsilioni I. Conti P. (2019). Mast Cells May Regulate The Anti-Inflammatory Activity of IL-37. Int. J. Mol. Sci. 20, 3701–3708. 10.3390/ijms20153701 PMC6696426 31362339

[B190] Turianova L. Lachova V. Svetlikova D. Kostrabova A. Betakova T. (2019). Comparison of cytokine profiles induced by nonlethal and lethal doses of influenza A virus in mice. Exp. Ther. Med. 18, 4397–4405. 10.3892/etm.2019.8096 31777543 PMC6862669

[B191] Valent P. Akin C. Hartmann K. George T. I. Sotlar K. Peter B.. (2017). Midostaurin: a magic bullet that blocks mast cell expansion and activation. Ann. Oncol. 28, 2367–2376. 10.1093/annonc/mdx290 28945834 PMC7115852

[B192] Van Rhee F. Wong R. S. Munshi N. Rossi J. F. Ke X. Y. Fossa A.. (2014). Siltuximab for multicentric Castleman’s disease: a randomised, double-blind, placebo-controlled trial. Lancet Oncol. 15, 966–974. 10.1016/S1470-2045(14)70319-5 25042199

[B193] Venkatesan S. Myles P. R. Leonardi-Bee J. Muthuri S. G. Al Masri M. Andrews N.. (2017). Impact of Outpatient Neuraminidase Inhibitor Treatment in Patients Infected With Influenza A(H1N1)pdm09 at High Risk of Hospitalization: An Individual Participant Data Metaanalysis. Clin. Infect. Dis. 64, 1328–1334. 10.1093/cid/cix127 28199524 PMC5411393

[B194] Verhoeven D. Perry S. (2017). Differential mucosal IL-10-induced immunoregulation of innate immune responses occurs in influenza infected infants/toddlers and adults. Immunol. Cell Biol. 95, 252–260. 10.1038/icb.2016.91 27629065

[B195] Vincent A. Awada L. Brown I. Chen H. Claes F. Dauphin G.. (2014). Review of influenza A virus in swine worldwide: a call for increased surveillance and research. Zoonoses Public Health 61, 4–17. 10.1111/zph.12049 23556412

[B196] Walter M. R. (2014). The molecular basis of IL-10 function: from receptor structure to the onset of signaling. Curr. Top. Microbiol. Immunol. 380, 191–212. 10.1007/978-3-662-43492-5_9 25004819 PMC4489423

[B197] Wang S. Z. Hallsworth P. G. Dowling K. D. Alpers J. H. Bowden J. J. Forsyth K. D. (2000). Adhesion molecule expression on epithelial cells infected with respiratory syncytial virus. Eur. Respir. J. 15, 358–366. 10.1034/j.1399-3003.2000.15b23.x 10706505

[B198] Wang L. Brown J. R. Varki A. Esko J. D. (2002). Heparin’s anti-inflammatory effects require glucosamine 6-O-sulfation and are mediated by blockade of L- and P-selectins. J. Clin. Invest. 110, 127–136. 10.1172/JCI0214996 12093896 PMC151027

[B199] Wang Z. Lai Y. Bernard J. J. Macleod D. T. Cogen A. L. Moss B.. (2012a). Skin mast cells protect mice against vaccinia virus by triggering mast cell receptor S1PR2 and releasing antimicrobial peptides. J. Immunol. 188, 345–357. 10.4049/jimmunol.1101703 22140255 PMC3244574

[B200] Wang Z. Macleod D. T. Di Nardo A. (2012b). Commensal bacteria lipoteichoic acid increases skin mast cell antimicrobial activity against vaccinia viruses. J. Immunol. 189, 1551–1558. 10.4049/jimmunol.1200471 22772452 PMC3411879

[B201] Webster R. G. Bean W. J. Gorman O. T. Chambers T. M. Kawaoka Y. (1992). Evolution and ecology of influenza A viruses. Microbiol. Rev. 56, 152–179. 10.1128/MR.56.1.152-179.1992 1579108 PMC372859

[B202] Wiernik P. H. (2010). FLT3 inhibitors for the treatment of acute myeloid leukemia. Clin. Adv. Hematol. Oncol. 8, 429–436, 444. 20733555

[B203] Xing Z. Gauldie J. Cox G. Baumann H. Jordana M. Lei X. F.. (1998). IL-6 is an antiinflammatory cytokine required for controlling local or systemic acute inflammatory responses. J. Clin. Invest. 101, 311–320. 10.1172/JCI1368 9435302 PMC508569

[B204] Xing Z. Harper R. Anunciacion J. Yang Z. Gao W. Qu B.. (2011). Host immune and apoptotic responses to avian influenza virus H9N2 in human tracheobronchial epithelial cells. Am. J. Respir. Cell Mol. Biol. 44, 24–33. 10.1165/rcmb.2009-0120OC 20118223 PMC3159084

[B205] Yang M. L. Wang C. T. Yang S. J. Leu C. H. Chen S. H. Wu C. L.. (2017). IL-6 ameliorates acute lung injury in influenza virus infection. Sci. Rep. 7, 43829. 10.1038/srep43829 28262742 PMC5338329

[B206] Yi N. Y. Newman D. R. Zhang H. Morales Johansson H. Sannes P. L. (2015). Heparin and LPS-induced COX-2 expression in airway cells: a link between its anti-inflammatory effects and GAG sulfation. Exp. Lung Res. 41, 499–513. 10.3109/01902148.2015.1091053 26495958 PMC4962551

[B207] Yu X. Zhang X. Zhao B. Wang J. Zhu Z. Teng Z.. (2011). Intensive cytokine induction in pandemic H1N1 influenza virus infection accompanied by robust production of IL-10 and IL-6. PloS One 6, e28680. 10.1371/journal.pone.0028680 22174866 PMC3235144

[B208] Zarnegar B. Mendez-Enriquez E. Westin A. Soderberg C. Dahlin J. S. Gronvik K. O.. (2017). Influenza Infection in Mice Induces Accumulation of Lung Mast Cells through the Recruitment and Maturation of Mast Cell Progenitors. Front. Immunol. 8, 310. 10.3389/fimmu.2017.00310 28382037 PMC5360735

[B209] Zarnegar B. Westin A. Evangelidou S. Hallgren J. (2018). Innate Immunity Induces the Accumulation of Lung Mast Cells During Influenza Infection. Front. Immunol. 9, 2288. 10.3389/fimmu.2018.02288 30337928 PMC6180200

[B210] Zhao W. Gomez G. Yu S. H. Ryan J. J. Schwartz L. B. (2008). TGF-beta1 attenuates mediator release and de novo Kit expression by human skin mast cells through a Smad-dependent pathway. J. Immunol. 181, 7263–7272. 10.4049/jimmunol.181.10.7263 18981148 PMC2645416

[B211] Zhou F. Zhu C. L. Niu Z. L. Xu F. X. Song H. Liu X. H. (2019). Influenza A virus inhibits influenza virus replication by inducing IL-37. J. Clin. Lab. Anal. 33, e22638. 10.1002/jcla.22638 30098064 PMC6430342

[B212] Zou Q. Wu B. Xue J. Fan X. Feng C. Geng S.. (2014). CD8+ Treg cells suppress CD8+ T cell-responses by IL-10-dependent mechanism during H5N1 influenza virus infection. Eur. J. Immunol. 44, 103–114. 10.1002/eji.201343583 24114149 PMC4165276

